# The Impact of Volatile and Non-Volatile Co-Extracted Matrix Components on the Reproducible Residue Analysis of Pesticides Using GC-MS/MS

**DOI:** 10.3390/molecules31091449

**Published:** 2026-04-27

**Authors:** Abd-Alrahman H. Abo-Gida, Al-Thabiani Aziz, Muhammed Issa, Sherif M. Taha, Amadeo R. Fernández-Alba

**Affiliations:** 1 Central Laboratory of Residue Analysis of Pesticides and Heavy Metals in Foods, Agricultural Research Center, Dokki, Giza P.O. Box 12311, Egypt; abdelrahmanabogaida@gmail.com (A.-A.H.A.-G.); muhammad.issa@qcap-egypt.com (M.I.); 2 Department of Biology, Faculty of Science, University of Tabuk, Tabuk 71491, Saudi Arabia; aalthbyani@ut.edu.sa; 3 European Union Reference Laboratory for Pesticide Residues in Fruit & Vegetables, Department of Chemistry and Physics, University of Almería, 04120 Almería, Spain; amadeo@ual.es

**Keywords:** pesticide residue, reproducible analysis, GC-MS/MS maintenance, strawberry, dry mint, Natural Analyte Protectant

## Abstract

This study presents a novel approach for optimizing GC-MS/MS performance and ensuring the reproducibility of pesticide residue analysis across diverse food matrices. Analysis of thermally treated (100–280 °C) extracts using GC-MS (scan mode) and FTIR revealed that strawberry and dry mint contain significantly higher concentrations of non-volatile co-extractives of varying chemical natures compared to fennel seeds. It was further elucidated that polar non-volatile co-extractives exhibit a more pronounced negative impact on analytical performance. Consequently, a synergistic approach was developed for strawberry analysis, combining end-column back-flushing with the application of fennel extract as a Natural Analyte Protectant (NAP). For dry mint, optimal results were achieved through a different approach: standard forward carrier gas cleaning combined with a fivefold sample dilution. The developed protocols enabled the efficient analysis of 195 pesticides in strawberries, all achieving LOQs of 0.01 mg/kg. Results demonstrated high precision (RSD < 3% for most analytes) and excellent recoveries (90–110%) at 0.01 and 0.05 mg/kg. Furthermore, 154 and 186 pesticides were successfully validated in dry mint with LOQs of 0.01 and 0.05 mg/kg, respectively. This research demonstrates that efficient column cleaning can be achieved through either back-flushing or the same forward-flow of the carrier gas, depending on whether the non-volatile co-extractives are polar or non-polar. Finally, ethyl acetate (EtOAc) fennel extract is introduced as a highly effective NAP, which is especially advantageous for samples lacking endogenous volatile components while simultaneously containing high concentrations of polar co-extractives. Pesticide residue monitoring was applied for 20 commercial samples, demonstrating high sensitivity. While strawberry samples exhibited excellent regulatory compliance and a total absence of chlorpyrifos, herbal matrices showed a higher chemical burden characterized by multi-residue co-occurrence and MRL exceedances.

## 1. Introduction

The routine multi-residue analysis of pesticides in food is usually accomplished using liquid chromatography and gas chromatography coupled with tandem mass spectrometry (LC-MS/MS and GC-MS/MS). A primary challenge in these analyses is the matrix effect, which significantly impacts data accuracy and precision [1]. In GC-MS/MS, pesticides prepared in pure solvents often exhibit lower responses compared to those in matrix-matched samples. This phenomenon occurs because pesticides in pure solvents are susceptible to adsorption onto the active sites of the GC-MS system [2]. However, co-extracted matrix components can enhance sensitivity by masking these active sites, thereby ensuring more consistent results, a phenomenon widely recognized as matrix-induced signal enhancement. It was previously reported that [3] pesticide residue analysis in high-water-content commodities, such as apples and grapes, often exhibits a strong signal enhancement, even though excessive co-extractives can elevate background noise and may hinder the detection of low concentrations [4], as seen in spelt kernel and sunflower seed samples [3]. To mitigate these effects, various cleanup sorbents, including primary secondary amine (PSA), graphitized carbon black (GCB), Enhanced Matrix Removal (EMR), and others, are employed to selectively remove interferences without compromising pesticide recovery [5,6,7]. In a comparative assessment across several food samples (spinach, orange, avocado, salmon, and bovine liver), PSA was identified as the optimal sorbent for analytical performance, while Zr-based dSPE was found to provide the highest cleanup capacity [8].

To reduce the concentration of co-extracted matrix components, previous studies have utilized less polar solvents, such as ethyl acetate (EtOAc) [9] or a solvent mixture of EtOAc: n-hexane [10], rather than the most commonly used extraction solvent, acetonitrile. Alternatively, some methodologies employ a solvent exchange step, replacing the acetonitrile, after pesticide extraction from the sample, with less polar solvents such as isooctane [11], acetone [12], a mixture of n-hexane: acetone [13,14], EtOAc [15], and toluene [16] before the GC-MS/MS injection. Such solvent exchange not only allows for a broad-scope extraction of pesticides using acetonitrile but also minimizes matrix interference through differential solubility and the adsorption of polar co-extracted components onto the surface of the used glass vessels. However, this evaporation step is time-consuming, increases the overall analysis costs, and elevates the risk of sample contamination. Consequently, applying the same amount of PSA cleanup to a smaller volume of the acetonitrile sample extract (3–5 mL) [17] provides a more practical analytical solution. Neutralizing the acetonitrile sample extract following PSA treatment is essential to ensure the stability of base-labile pesticides [18]. This approach effectively reduces matrix co-extractives while remaining compatible with a standard 1 µL direct injection of the acetonitrile extract.

To mitigate the disparity between solvent-based standards and real samples, a matrix-matched calibration approach is commonly employed [19]. Furthermore, adding specific analyte protectants, such as sugars and sugar lactones, to sample extracts has been reported to shield analytes from active sites of the used GC-MS system [20,21]. On the other hand, some previous studies have explored Natural Analyte Protectants (NAPs) derived from plant matrices, including pepper leaf [22], cucumber [23], orange [19], and fennel seeds [17], to achieve robust protection with fewer long-term adverse effects on the GC-MS/MS performance. To maintain the ruggedness of the GC-MS/MS system and reduce the frequency of preventive maintenance, inlet liners containing glass wool [24] or specific geometries (e.g., baffled or tapered) [25] are preferred. Additionally, column cleaning protocols are essential for maintaining data reproducibility. Common strategies include thermal cleaning using the same forward carrier gas flow [26,27] or backflushing (reversing the helium flow after eluting the last targeted pesticide). Both methods were conducted at high temperatures (300–310 °C) to facilitate the elution of non-volatile co-extracted components [28]. However, the reproducibility of GC-MS/MS is frequently compromised by the accumulation of matrix co-extractives, particularly in complex matrices such as oranges [29], strawberries [17], spices [30], and herbal plants [18,28]. While these matrices may introduce high concentrations of co-extractives that typically provide a transient masking effect, they eventually contaminate the system and preclude the achievement of low detection limits [1]. Consequently, isotopically labeled internal standards are used to effectively compensate for the negative impact of the matrix effect [31]. However, the high cost of isotopically labeled standards often limits their widespread adoption in large-scale routine analysis.

This study aims to characterize and identify matrix co-extractives components in two distinct food matrices, fresh strawberry and dry mint, to evaluate their impact on the reproducibility of pesticide residue analysis using GC-MS/MS. To provide an in-depth exploration of the effects of such co-extracts and distinguish their volatile and non-volatile constituents, mass scan analyses were conducted. The selected thermal conditions, 100 °C and 280 °C, were chosen to simulate the initial and final stages of the GC oven temperature program, as well as the GC inlet temperature. Complementary infrared (IR) spectroscopy was employed to elucidate the chemical nature of these extracts further. Finally, column cleaning protocols, utilizing either the same carrier gas flow or reversing the flow, were optimized to enable a long-term method stability and system robustness. The optimized method was validated in strawberries at two concentration levels of 0.01 and 0.05 mg/kg, and in dry mint at three concentrations of 0.01, 0.05, and 0.25 mg/kg. All validation procedures were performed in strict accordance with the SANTE/11312/2021 v2026 EU guidance document [32]. The optimized analytical approaches were applied to perform a monitoring study of pesticide residues in 20 commercial samples of strawberry, dry mint, and moringa.

## 2. Result and Discussion

### 2.1. Analysis the Matrix Components of the Tested Commodities

By comparing the Total Ion Chromatograms (TICs) of the analyzed samples across the three specified mass ranges, it was observed that the majority of eluted compounds in the extracts fall within the 50 to 250 Da range. No significant eluted peaks corresponding to higher masses (>250 Da) were detected. Furthermore, the narrower mass range of 50–250 Da provided superior sensitivity for the relevant eluted peaks compared to the wider mass range of 50–500 Da. Consequently, the TICs within the 50–250 Da range will be the primary focus of the discussion below.

#### 2.1.1. Analysis of Acetonitrile Extracted Matrix Components, Thermally Untreated

**Figure 1:**
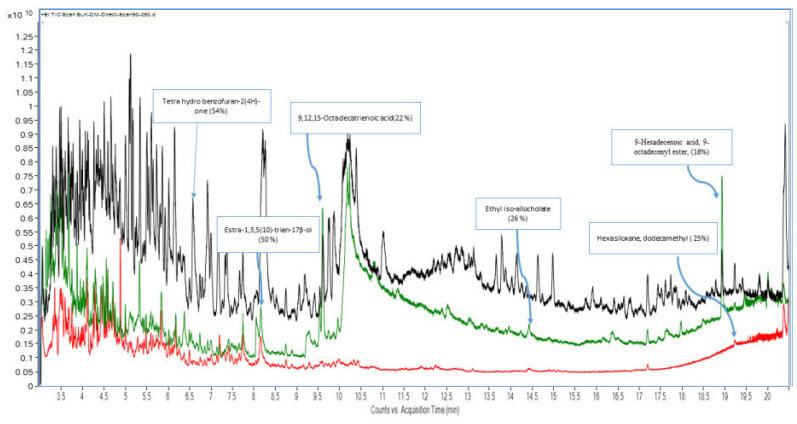
Total ion chromatograms (GC−MS scan, acquired in scan mode of m/z 50–250) of thermally untreated acetonitrile extracts of dry mint (black), fennel seed (green), and strawberry (red), with some identified natural compounds and their matching probability using NIS library.

As expected, scan chromatograms of dry mint and fennel seed showed a high abundance of co-extracted components throughout the entire run time, as depicted in Figure 1. Most of these eluting volatile matrix components in these herbs are long-chain alcohols, ketones, acids, and terpenes. There are also late-eluting peaks related to higher molecular weight volatile extracts that correspond to triterpenoids and retinoids (Figure 1). Such heavy late-eluting volatile extracts were present in dry mint at much higher levels than in fennel seed. Most of the volatile compounds act as NAPs when analyzing pesticides in these matrices. Conversely, the strawberry scan showed significantly fewer peaks, especially after 8 min. The absence of NAPs leaves the target pesticides in the strawberry exposed to the active sites of the GC-MS system, resulting in severe adsorption and various subsequent negative issues that affect the accuracy and precision of the results.

#### 2.1.2. Analysis of Acetonitrile Extracted Matrix Components After Thermal Heating at 100 °C

Thermal treatment of the acetonitrile extracts at 100 °C for 2 h was conducted to simulate the initial temperature of the GC oven temperature program. This treatment resulted in a viscous, oily residue, particularly for dry mint and strawberry (Supplementary Figure S1(B1,C1)). Notably, these residues were not completely resolubilized in acetonitrile for the subsequent GC-MS scan analysis (Supplementary Figure S1(B2,C2)). The formation of such a dense, oily layer suggests that these semi- to non-volatile components undergo rapid adsorption onto the column’s stationary phase, where they are subsequently carbonized during the high-temperature stages of the applied oven program. The MS scan analysis of the solubilized, thermally treated residues for both mint and fennel (Supplementary Figure S2) showed that they still retained a large number of volatile NAPs. This reveals that dry mint possesses a dual-character matrix: it contains volatile components that act as effective NAPs throughout the entire analytical run, while simultaneously carrying heavy, oily components that contribute to physical system contamination. In contrast, the redissolved thermally treated residue of the strawberry was devoid of protective co-extracted volatile components. Therefore, this result confirms that strawberry induces primarily negative matrix effects, which are attributed to the high load of non-volatile, oily residues remaining after thermal treatment.

#### 2.1.3. Analysis of Acetonitrile Extracted Matrix Components After Thermal Heating at 280 °C

The extracts of the tested samples were subjected to an additional thermal treatment at 280 °C, simulating the extreme temperatures experienced within the GC inlet liner and the GC column during the final stages of the oven temperature program. This treatment yielded completely dry solid residues, with a markedly higher mass observed for strawberry and dry mint.

**Figure 2:**
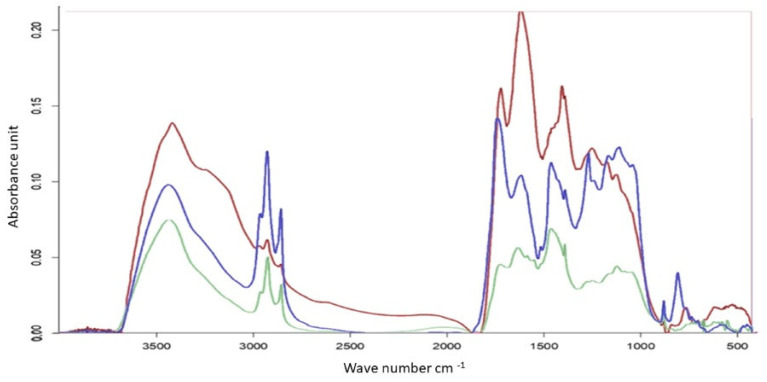
IR spectra of the re-dissolved acetonitrile extracts after their thermal treatment at 280 °C for strawberry (red), fennel seed (blue), and dry mint (green).

FTIR analysis of these solids (Figure 2) revealed that strawberry possessed the highest intensity band between 3200 and 3600 cm^−1^, including a unique broad peak at 3200 cm^−1^ that indicates high sugar and pectin content characterized by intense intermolecular hydrogen bonding. In contrast, dry mint and fennel displayed sharp aliphatic peaks at 2800 and 2900 cm^−1^, suggesting the presence of stable lipids or waxes that were absent in strawberry. Strawberry also exhibited the highest intensity across the 800–1900 cm^−1^ region, confirming the formation of a dense, oxygenated carbonaceous phase.

**Figure 3:**
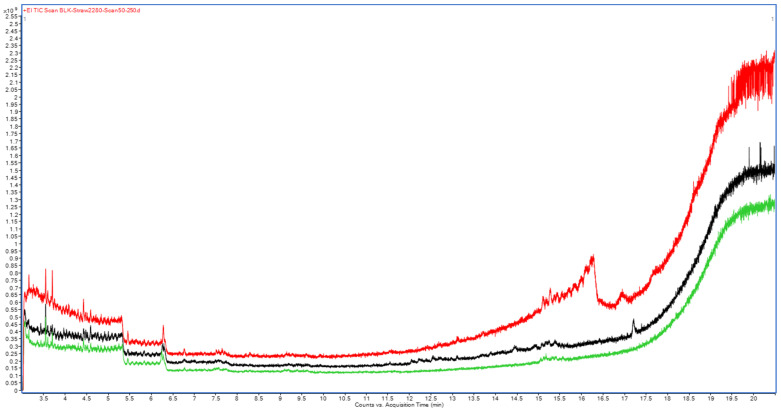
Total ion chromatograms (GC−MS scan) of acetonitrile extracts subjected to thermal stress at 280 °C for 2 h of strawberry (red), dried mint (black), and fennel seed (green).

Conversely, MS scan analysis of these redissolved solid residues (Figure 3) showed an almost complete absence of volatile co-extracted peaks across all tested commodities. However, the strawberry extract exhibited a significantly elevated baseline, indicating notable GC-MS system contamination and the accumulation of non-volatile matrix components. The adsorption of these non-volatile co-extracted components, initially as oily layers at 100 °C and through subsequent carbonization at higher temperatures, leads to a marked deterioration of the GC-MS system. Consequently, a higher frequency of column trimming and inlet maintenance is required to sustain the analytical efficiency and ruggedness of the GC system when analyzing these complex matrices

#### 2.1.4. Analysis of Fennel Extracted Matrix Components Using Acetonitrile and Ethyl Acetate

Both the acetonitrile and EtOAc extracts of fennel seed have a large number of co-extracted components throughout the TIC, as shown in Supplementary Figure S3. This is in alignment with the higher percentage of phenolic components in fennel, particularly estragole [33]. However, the EtOAc extract of fennel possesses a higher number of volatile extracted compounds that can protect the analyzed target pesticides in strawberry throughout the entire analytical run compared to the acetonitrile extract. This may be attributed to the greater solubility of these extracts, phenols, in EtOAc than in acetonitrile. Since fennel seed exhibited a lower baseline after thermal treatment at 280 °C (Figure 3) and yielded the lowest volume of oily, insoluble residue after thermal treatment at 100 °C, it is considered the most suitable matrix source to be used as a NAP.

#### 2.1.5. Evaluation of Forward Gas Flow Versus Backflush Cleaning

**Figure 4:**
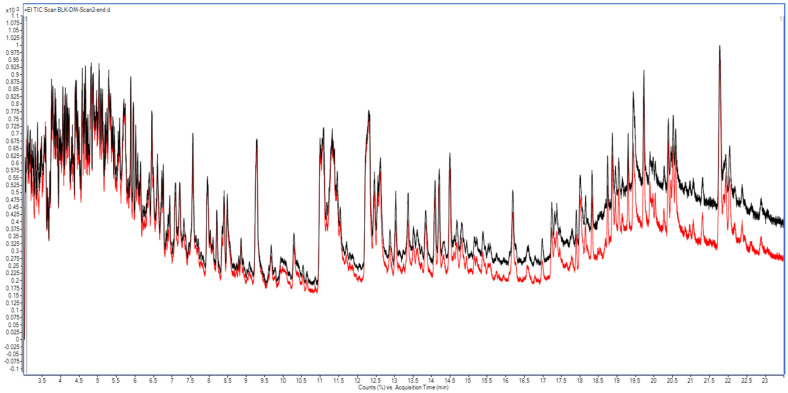
Total ion chromatograms (GC−MS scan) of dry mint extract under the same gas flow with an additional 3 min column cleaning step. The red baseline represents the initial scan analysis, and the black baseline represents the scan analysis after 11 consecutive injections.

In the dry mint matrix, repeated scan analysis revealed that the MS baseline became significantly elevated (often higher than the first injection, as expected), regardless of whether the same forward gas flow (SF) or backflush (BF) was used, as shown in Figure 4 and Supplementary Figure S4, respectively. Comparing these TICs, only a slight baseline increase was observed with SF cleaning following repeated dry mint injections. This may be attributed to the presence of higher molecular weight volatile residues (triterpenoids and retinoids) and nonvolatile (waxes and lipids) in dry mint, which do not readily migrate when the carrier gas flow is reversed during back-flushing. This confirms that dry mint possesses a complex herbal matrix characterized by high concentrations of volatile components, alongside a substantial content of non-polar, non-volatile residues.

**Figure 5:**
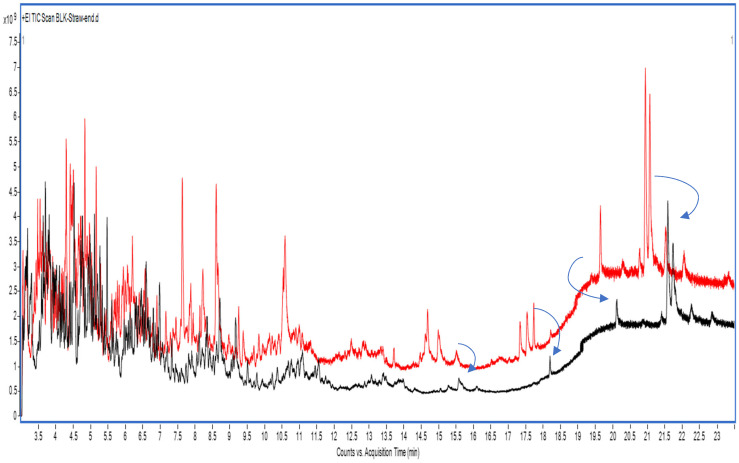
Total ion chromatograms (GC−MS scan) of strawberry extract (without fennel as NAP) under the same forward gas flow with an additional 3 min column cleaning step. The red baseline represents the initial scan analysis, and the black baseline represents the scan analysis after 11 consecutive injections.

**Figure 6:**
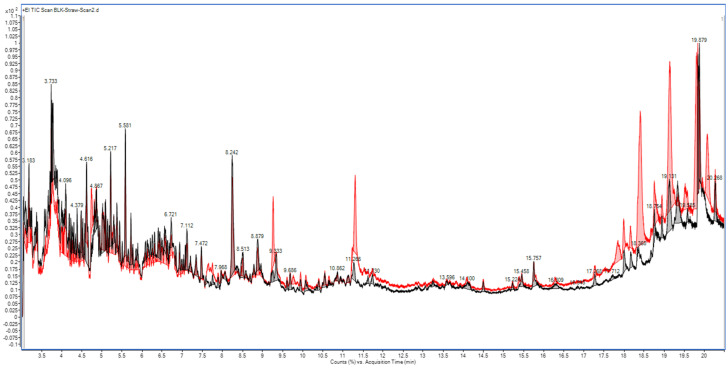
Total ion chromatograms (GC−MS scan) of strawberry extract with fennel as NAP under backflush gas flow during an additional 3 min column cleaning step. The red baseline represents the initial scan analysis, and the black baseline represents the scan analysis after 11 consecutive injections.

In the strawberry matrix, without mixing with EtOAc fennel extract as NAPs, the repeated scan analysis revealed that the MS baseline was markedly lowered after cleaning the column using SF and BF, as shown in Figure 5 and Supplementary Figure S5, respectively. The same results were obtained for the strawberry matrix, which was mixed with the NAP_S_ at both BF and SF conditions, as shown in Figure 6 and Supplementary Figure S6, respectively. Furthermore, a significant peak delay occurred for most co-extracted matrix components, particularly under SF, as shown in Figure 5. This unexpected decrease in the MS baseline following repeated strawberry injections stems from the adsorption of non-volatile co-extractives of polar nature, specifically carbohydrates and pectins, onto the glass liner and the fore-section of the analytical column. With repeated injections and the high thermal stress of the GC-MS system, these polar non-volatile matrix components are pyrolyzed. The resulting pyrolyzed components may have a higher polar activity and, therefore, exert a marked negative effect on eluted components, reducing both their number and sensitivity, and significantly delaying their elution, especially in the case of SF (Figure 5). Furthermore, it was elucidated that the polar non-volatile strawberry co-extractives also interact with the GC column stationary phase. This interaction was evidenced by the presence of dodeca-methyl-hexa-siloxane in the TIC of the strawberry extract, indicating stationary phase bleeding induced by the matrix (Figure 1). Such bleeding interactions with the stationary phase result in a TIC with an unexpected lower baseline (Figure 5). Therefore, the previously reported usage of sugars and sugar lactone as APs [20,34] is not an acceptable approach for routine repeated GC-MS/MS injections. On the other hand, the BF strategy demonstrated its effectiveness in eliminating these polar, non-volatile components. Therefore, cleaning a smaller volume of the strawberry acetonitrile extract with an equivalent percentage of PSA is essential, especially when directly injecting the acetonitrile extract into the GC-MS/MS system [17]. Furthermore, it is suggested that at least a twofold sample dilution of strawberry (using a 5 g sample weight) is advantageous for the same reason. By BF, the system minimized these non-volatile active residues within a short run time and maintained superior overall system performance (Figure 6). A similar beneficial effect of using a BF system was previously reported for the removal of non-volatile hydrocarbon carryover [35].

### 2.2. Method Precision and Reproducibility

**Figure 7:**
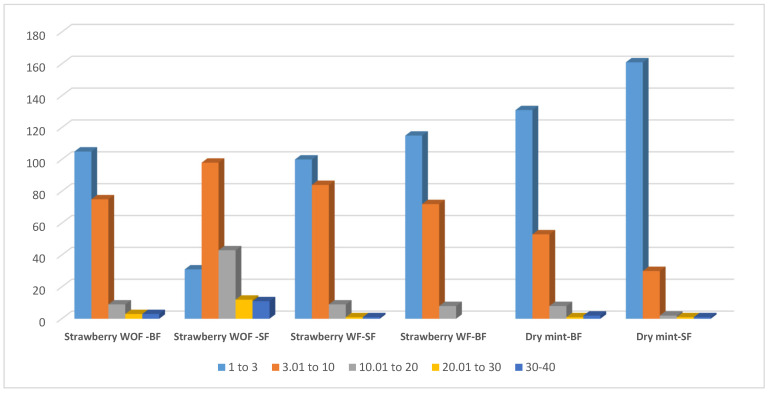
Relative standard deviation of the repeated injection (*n* = 11) of the same matched calibration point 0.05 mg/L in strawberry with fennel extracts as NAPs (WF) and without (WOF), using column cleaning by the same gas flow (SF) and back flush (BF). RSD of the repeated injection (*n* = 10) of the same matched calibration point 0.05 mg/L in dry mint using column cleaning by the same gas flow (SF) and back flush (BF).

The reproducibility analysis of the target pesticides was tested through repeated injections of a matrix-matched calibration point at 0.05 mg/L (*n* = 11, with considering the first one as a cal. point) in strawberry and dry mint under various matrix and cleaning conditions. Strawberry exhibited the lowest reproducibility and high RSD values when applying SF column cleaning. Better results were obtained when applying the BF cleaning (Figure 7). This result is in alignment with the above scan analyses. It also confirms that polar, non-volatile components are not easily removed through the SF column cleaning at high temperatures. Instead, these polar matrices and their degradation products may further interact with the stationary phase of the column during their slow migration through the long column path under extended thermal holds with SF. In contrast, the BF cleaning resulted in reproducible pesticide residue analysis in strawberries during repeated injections by a fast physical removal of such polar non-volatile components in a short path. Furthermore, when using fennel extract as an NAP with strawberry, improved reproducibility was achieved. Overall, the highest precision was achieved by combining BF with the application of an EtOAc fennel extract as an NAP. This synergistic approach yielded significantly superior, reproducible results (RSD < 3%) for 115 pesticides in the repeated injections of matrix-matched calibration standards in strawberry (Figure 7). Meanwhile, dry mint showed a higher number of reproducible results (131 and 161 pesticides using BF and SF column cleaning, respectively). This result confirms the above suggestion, based on the mass scan analyses, that the higher molecular weight volatile and nonvolatile residues in dry mint do not readily migrate when the carrier gas flow is reversed, during BF. These results show that the non-polar, non-volatile matrix is intrinsically more stable and places a lower burden on the GC–MS system than the polar, non-volatile strawberry matrix, which promotes greater system deterioration.

### 2.3. Validation of the Optimized Method

**Figure 8:**
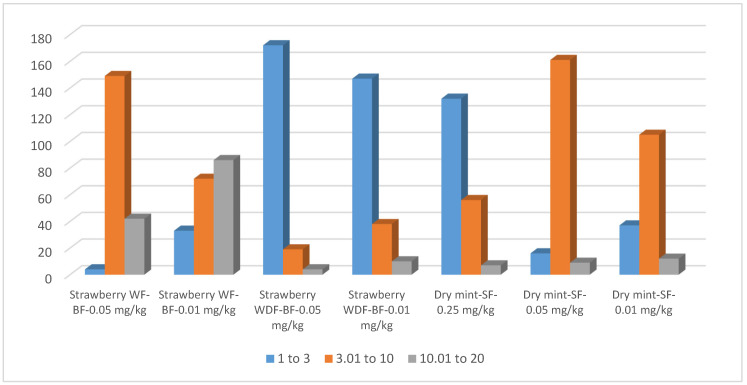
Relative standard deviation of the intra-repeated validation measurement of strawberry with back flush cleaning (BF) and mixing with fennel (WF) and concentrated fennel (WDF) extracts as NAPs, all with applying back flush (BF) cleaning. RSD intra-repeated validation measurement in dry mint with column cleaning by the same gas flow (SF) at three concentration levels.

Validation at 0.01 and 0.05 mg/kg in strawberry with fennel as NAP showed that precision was improved at the higher level, with most pesticides achieving RSDs below 10, as shown in Figure 8. However, dry mint remained the more stable matrix, yielding a higher number of pesticides with RSD values below 3, while the high concentration of volatile and non-polar co-extractives in dry mint resulted in a high Limit of Quantification (LOQ) of 0.05 mg/kg (0.01 mg/L) and 0.25 mg/kg (0.05 mg/L) for thirty-two and nine pesticides, respectively (Supplementary Table S1). However, the method remained highly effective for the majority of analytes. Specifically, 154 pesticides were successfully determined in dry mint with LOQs of 0.01 mg/kg (0.002 mg/L).

**Figure 9:**
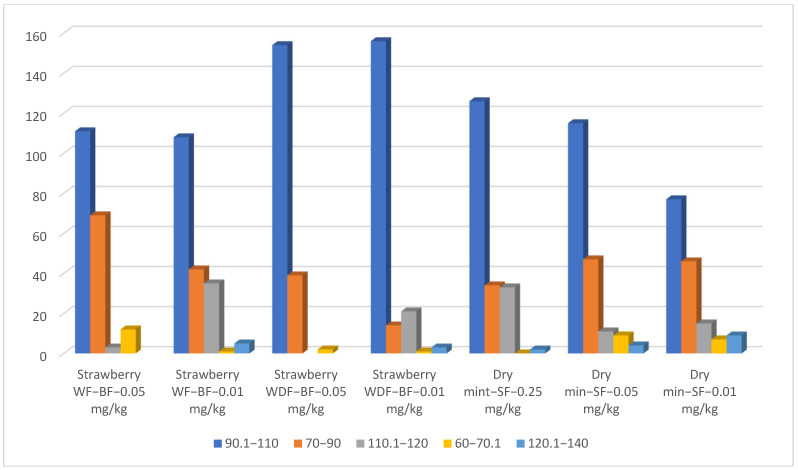
Recovery of the intra-repeated validation measurement of strawberry with back flush cleaning (BF) and mixing with fennel (WF) and concentrated fennel (WDF) EtOAc extracts as NAPs at two different fortification levels. Recovery of intra-repeated validation measurement in dry mint with column cleaning by the same gas flow (SF) at three fortification levels.

Additional validation was performed in strawberry using a concentrated EtAOc fennel extract as NAP. This approach led to a significant improvement in both precision and accuracy, as seen in Figure 8 and Figure 9. It is suggested that the concentrated NAPs of the EtAOc fennel extract provided a superior masking effect not only to silanol groups of the GC-MS system but also to deactivate the huge polar nonvolatile components of the strawberry, resulting in highly reproducible intra-day measurements.

The matrix effects and linearity measurements (correlation coefficients, R^2^) are summarized in Supplementary Table S2. The optimized method demonstrated an improved linearity through concentrations of 0.002 to 0.5 mg/L, with R^2^ values exceeding 0.95 for the majority of the target pesticides, confirming the method’s analytical efficiency. Notably, the matrix effect profiles for both dry mint and strawberry became comparable following the addition of concentrated fennel extract (NAP). Specifically, matrix effects in the strawberry matrix ranged from −99.96% to −71.65%, while dry mint exhibited a range of −102% to −72.96%, both relative to the green bean matrix. These results suggest that while matrix suppression remains significant compared to the green bean matrix, the natural volatile components inherent to dry mint, along with the ethyl acetate (EtOAc) fennel extract added to the strawberry, effectively standardize the response of the targeted pesticides.

### 2.4. The Analysis of Real Market Samples

The concentration ranges of detected pesticide residues in the monitored market samples (strawberry, dry mint, and moringa matrices) and the number of contaminated samples are depicted in Table 1. These results indicate clear differences in pesticide use patterns and residue profiles across the studied matrices. Strawberry samples (*n* = 10) showed an overall favorable safety profile: although nine different pesticides were detected across the set, individual samples typically contained only a small number of residues. Typically, each analyzed strawberry sample contained only one to two residues. All detected concentrations remained significantly below the EU-established Maximum Residue Limits (MRLs). The most promising result was the complete absence of chlorpyrifos in all strawberry samples, which serves as a strong indicator of improved agricultural pesticide practices in Egypt. This reflects the successful transition of local farmers toward safer alternatives following its recent prohibition in Egypt by the National Food Safety Authority (NFSA) and the Central Administration for Agricultural Quarantine (CAPQ). Furthermore, the absence of co-existing pesticides with the same Mode of Action (MoA) within any single strawberry sample suggests a well-managed rotation strategy that effectively reduces the risk of developing pesticide resistance.

In contrast, the herbal matrices presented a more complex contamination profile characterized by frequent multi-residue co-occurrence and a higher chemical burden. Within the dry mint samples (*n* = 7), seven different pesticide residues were identified, including the detection of chlorpyrifos in four samples with a maximum concentration of 0.039 mg/kg, which exceeds the current EU MRL of 0.01 mg/kg. The simultaneous presence of atrazine, profenofos (0.010 mg/kg), l. Cyhalothrin (0.014 mg/kg) and cypermethrin in one dry mint sample highlight a potential risk for cumulative toxicological effects. Similarly, the moringa samples (*n* = 3) showed high herbicide frequencies, with atrazine reaching 100% occurrence, and one specific sample demonstrating a significant simultaneous detection of five pesticides: atrazine, chlorfenapyr, cypermethrin, oxyfluorfen, and l. cyhalothrin. Such high numbers of pesticide residues in herbal samples likely reflect the pooling of material from multiple fields, heterogeneous local agricultural practices, and, potentially, uncontrolled post-harvest applications during storage. Ultimately, the successful identification of these diverse pesticide classes across varying matrices confirm the robustness and high sensitivity of the developed GC-MS/MS method.

## 3. Materials and Methods

### 3.1. Chemicals and Reagents

Methanol and toluene were supplied by Supelco (Bellefonte, PA, USA), while acetonitrile and EtAOc were purchased from Fisher Scientific (Waltham, MA, USA). Formic acid was obtained from Carlo Erba (Milan, Italy). Ultrapure deionized water (DIW) with a resistivity of 18.2 MΩ·cm was prepared using a Milli-Q water purification system (Merck, Darmstadt, Germany). Ready-to-use QuEChERS salting out packets (Part Number: 5982-7650, containing MgSO_4_, NaCl, and citrate buffer salts) and dispersive solid-phase extraction (d-SPE) cleanup salts (Part Number: 5610-2129, containing MgSO_4_ and PSA) were purchased from Agilent Technologies (Santa Clara, CA, USA). Analytical-grade reference standards of 195 pesticides (purity > 98% for the majority) were obtained from Dr. Ehrenstorfer (Augsburg, Germany) and Sigma-Aldrich (Burlington, MA, USA). Individual stock standard solutions for the majority of the targeted pesticides were prepared in high-purity toluene and stored at −20 °C, as this solvent ensures optimal stability and minimal degradation of the analytical standards. Subsequently, several working standard mixtures were prepared in acetonitrile and stored at 4 °C.

### 3.2. Market Sample Collection

Twenty commercial samples (strawberry (*n* = 10), dry mint (*n* = 10), and moringa (*n* = 10)) were randomly collected from various local markets across the Cairo and Giza regions, Egypt. To ensure a representative laboratory sample, a minimum weight of 2 kg was collected for strawberries, while at least 250 g was obtained for each herbal matrix.

All samples were analyzed immediately upon arrival at the laboratory. The remaining portions of the strawberry homogenates were stored in a freezer at −20 °C for any further confirmatory analysis

### 3.3. Sample Processing and Preparation

The strawberry samples were thoroughly homogenized using a high-speed blender. The dry matrices were ground into a fine powder (particle size 160–200 µm) with 160 and 200 µm test sieves. Grinding included intermittent pauses to prevent heat buildup from friction, which could cause thermal degradation of sensitive pesticide residues. Strawberry samples were prepared following the standard QuEChERS protocol, involving extraction with acetonitrile and phase separation using a pre-packaged salting-out mixture. Next, 4 mL of extract was cleaned up and filtered through a 0.45 μm syringe filter, then neutralized with acidified acetonitrile containing 5% formic acid before GC-MS/MS injection [18]. A similar sample preparation method was used for the dry mint and fennel seed samples. Specifically, a two-gram sample was weighed and then hydrated with 10 mL of deionized water, providing a fivefold dilution. The mixture was vortexed for one minute and shaken for five minutes to ensure full hydration. The same QuEChERS procedure as for the strawberries was then followed. For the NAP: Fennel extracts, the procedure was similar, but acetonitrile was replaced by EtOAc as the extraction solvent. Two different sample weights (2 g and 4 g) were tested, resulting in fivefold and 2.5-fold dilutions, respectively. The 4 g sample, referred to as ‘concentrated EtOAc fennel extract,’ was used throughout. To assess the protective effect of NAPs, 950 µL of the acetonitrile strawberry extract was mixed with 50 µL of either the standard or concentrated EtOAc fennel extract before GC-MS/MS injection.

### 3.4. Fourier-Transform Infrared Analysis

Fourier-transform infrared (FTIR) spectra were recorded using a Vertex 70 spectrometer (Bruker, Berlin, Germany), equipped with a diamond Attenuated Total Reflectance (ATR) accessory at the National Research Centre (NRC), Egypt. The accessory provided a penetration depth of approximately 2 µm. Spectra were acquired over the range of 4000–400 cm^−1^ at a resolution of 4 cm^−1^. Each spectrum was the result of 40 co-added scans, with background measurements taken against air using the same experimental parameters.

### 3.5. GC-MS/MS Instrumentation and Conditions

Gas chromatographic analysis was performed using an Agilent 7890B GC system coupled with a 7010B triple quadrupole mass spectrometer (Agilent Technologies, Santa Clara, CA, USA). Analyte ionization was achieved via electron ionization (EI), and separation was performed on an HP-5MS Ultra Inert capillary column (30 m × 0.25 mm, 0.25 µm; Agilent). High-purity helium (≥99.999) served as the carrier gas. The oven temperature program was as follows: initial temperature of 50 °C (hold for 0.7 min), increased to 170 °C at 60 °C/min (no hold), then to 270 °C at 7 °C/min (no hold), and finally to 310 °C at 20 °C/min. An additional three-minute hold was applied at this maximum temperature to clean the column after the elution of the last pesticide, deltamethrin, using the same forward or reverse carrier gas flow, SF or BF, respectively. These two clean-up approaches were tested and compared for their efficiency in providing reproducible pesticide residue analysis in strawberry and dry mint. The injection volume was 1 µL into a liner containing glass wool, with a constant inlet temperature of 280 °C. Each pesticide was analyzed via GC-MS/MS using at least two multiple reaction monitoring (MRM) transitions [17]. The MRM transitions, collision energies, and retention times of the target pesticides were depicted in Supplementary Table S3. Furthermore, GC-MS scans were performed using the first quadrupole as a mass guide for three distinct mass ranges: 50–500 Da, 50–250 Da and 250–500 Da.

### 3.6. Analysis of Volatile and Nonvolatile Matrix Components of the Tested Commodities

Several analyses were performed to identify the natural components of the tested samples and study their subsequent effect as co-extracted components on the reproducible residue analysis of target pesticides. The extracts of the tested samples (strawberry, fennel seeds, and dry mint), obtained via the different extraction procedures as mentioned above, were first analyzed using GC-MS in scan mode. Furthermore, vials containing strawberry extract (with and without EtOAc fennel extract) and dry mint extract were repeatedly injected (*n* = 11, considering the first one as a calibration point) into the GC-MS scan analysis using two different column cleaning configurations (SF and BF). In addition, the acetonitrile extracts (10 mL) of the tested samples were subjected to two different thermal treatments at 100 °C and 280 °C for 2 h, reconstituted to the original volume with acetonitrile, and analyzed also by GC-MS scan. Furthermore, the remaining solid residue following thermal treatment at 280 °C was collected and analyzed by IR spectroscopy.

### 3.7. Method Validation

The optimized analysis protocols were validated for accuracy and precision through recovery and repeatability measurements. Validation was conducted by fortifying blank samples (*n* = 6) with 195 pesticides. For the strawberry, fortification levels were set at 0.01 and 0.05 mg/kg. For dry mint, levels were set at 0.01, 0.05, and 0.25 mg/kg (corresponding to extract concentrations of 0.002, 0.01, and 0.05 mg/L, respectively).

Linearity was evaluated using five-point matrix-matched calibration curves prepared in blank green beans, strawberry and dry mint extracts at concentrations of 0.002, 0.01, 0.05, 0.1, and 0.5 mg/L.

Matrix effects (ME) were evaluated by comparing the analytical responses of the targeted pesticides in the optimized strawberry and dry mint extracts to those in a green bean reference matrix, according to the following equation

*ME* % = ((*Slope of pesticide clibaration in A*)/(*Slope of pesticide clibaration in B*) – 1) × 100

*A*: blank acetonitrile sample extract (dry mint and strawberry mixed with the concentrated NAP).

*B*: blank acetonitrile green beans extract.

## 4. Conclusions

The negative impact of non-volatile co-extractives is frequently overlooked because they do not produce visible co-eluting peaks in triple quadrupole gas chromatography analysis. This study has demonstrated that thermal treatment at 100 °C reveals significant oily residues in both strawberry and dry mint. Treatment at 280 °C resulted in the highest residual mass for both matrices. GC-MS and FTIR analyses confirmed that strawberry extracts are mainly polar non-volatile compounds, present at significantly higher intensities than in dry mint or fennel. These polar non-volatile matrix components not only exert a direct negative impact on the peak shape and sensitivity of the targeted pesticides, but also accelerate the degradation of the stationary phase. A synergistic analytical approach was developed for pesticide residue analysis in strawberries. This approach combined back-flushing with the use of concentrated ethyl acetate (EtOAc) fennel extract as a Natural Analyte Protectant (NAP). This framework ensured high precision for 195 pesticides at the 0.01 mg/kg level. In contrast, volatile-rich matrices like dry mint provide a degree of natural protection. However, their excessive volatile and non-polar non-volatile components raised the detection limits for nine pesticides. After a fivefold dilution and extended column cleaning using forward carrier gas flow, 154 pesticides could be detected at 0.01 mg/kg. These results confirm that column cleaning after analysis of samples containing non-volatile components, whether polar or non-polar, should be accomplished by maintaining the runtime at a higher temperature. The carrier gas should be reversed or maintained in forward flow, respectively. Furthermore, the used herbal-based NAPs provided a superior masking effect. They deactivated the active silanol groups within the GC-MS system and mitigated the impact of the polar non-volatile components of the strawberry matrix. The application of the developed GC-MS/MS method to market samples successfully identified diverse pesticide classes, confirming its robustness for routine food safety monitoring. While strawberry samples demonstrated high compliance and a positive shift toward safer practices following the national ban on chlorpyrifos, the herbal matrices (dry mint and moringa) revealed a higher chemical burden characterized by frequent multi-residue co-occurrence and MRL exceedances. Future studies should implement at least a twofold dilution for fresh samples high in polar non-volatile co-extractives, and a tenfold dilution for herbal samples high in nonpolar non-volatile co-extractives. Future studies utilizing high-resolution GC-MS (HRMS) systems may provide superior identification of both volatile and non-volatile co-extractives, allowing for a more in-depth examination of their specific effects on pesticide residue analysis.

## Figures and Tables

**Table 1:** Occurrence and concentration ranges of detected pesticide residues in monitored market samples (*n* = 20).

Matrix	Pesticide	Positive Samples (*n*)	Min (mg/kg)	Max (mg/kg)
Strawberry, (*n* = 10)	Diniconazole	1	<LOQ *	<LOQ *
	Metalaxyl	2	0.01	0.069
	Difenoconazole	2	0.011	0.012
	Pyridaben	1	0.014	0.014
	Pyrimethanil	1	0.031	0.031
	Fenazaquin	2	0.027	0.029
	Cyprodinil	1	0.014	0.014
	Boscalid	1	0.015	0.015
	L. Cyhalothrin	1	<LOQ *	<LOQ *
Dry Mint, (*n* = 7)	Atrazine	5	<LOQ *	0.02
	Chlorpyrifos	4	<LOQ *	0.039
	L. Cyhalothrin	1	0.014	0.014
	Cypermethrin	3	<LOQ *	0.01
	Profenofos	2	<LOQ *	0.01
	Propiconazol	1	<LOQ *	<LOQ *
	Pendimethalin	5	0.012	0.032
Moringa, (*n* = 3)	Atrazine	3	0.011	0.032
	Pendimethalin	2	0.015	0.017
	Oxyfluorfen	2	0.021	0.027
	L. Cyhalothrin	1	<LOQ *	<LOQ *
	Chlorfenapyr	1	0.03	0.03
	Cypermethrin	1	0.031	0.031

* <LOQ: The detected value is less than the Limit of Quantitation of the validated method (0.01 mg/kg for strawberry and dry mint).

## Data Availability

The data presented in this study are available within the article and its Supplementary Materials.

## Supplementary Materials

The following supporting information can be downloaded at: https://www.mdpi.com/article/10.3390/molecules31091449/s1, Figure S1: The thermally treated acetonitrile extracts (10 mL each) of fennel seeds, strawberry, and dry mint were prepared at 100 °C for 2 hours and after their redesolving with the same volume of acetonitrile as A1, B1, & C1, and A2, B2, and C2, respectively; Figure S2: The total ion chromatogram of the GC-MS scan analysis of acetonitrile extracts of dry mint (black), fennel seed (green), and strawberry (red) after their thermal treatment at 100 °C for 2 hours; Figure S3: The total ion chromatograms of the GC-MS scan analysis of acetonitrile and ethyl acetate extracts of fennel seed in blue and green, respectively, with the acetonitrile extract of strawberry in red; Figure S4: The total ion chromatograms of the GC-MS scan analysis of acetonitrile dry mint extract with back flush cleaning at first batch injection in red and that after repeated injection (n = 11) in black; Figure S5: The total ion chromatograms of the GC-MS scan analysis of acetonitrile strawberry extract (without mixing with fennel as NAP and with applying back flush) at the first batch injection in red and after repeated injection (n = 11) in black; Figure S6: The total ion chromatograms of the GC-MS scan analysis of acetonitrile strawberry extract (with mixing with fennel as NAP and with applying the same gas flow column cleaning) at first batch injection in red and that after repeated injection (n = 11) in black; Table S1: The result of the Intra-day measurements in strawberry and dry mint using the developed methods: Recovery (Rec, %) and relative standard deviation (RSD, %) values for the targeted pesticides at various fortification levels; Table S2: Linearity (Correlation Coefficient, R2) and Matrix Effect (ME, %) for the targeted pesticides in strawberry and dry mint extracts; Table S3: The mass transitions (precursor and product ions), collision energies (CE), and retention times (RT) for the targeted pesticides.
