# Supplementary material for: The Impact of Volatile and Non-Volatile Co-Extracted Matrix Components on the Reproducible Residue Analysis of Pesticides Using GC-MS/MS
Source: Molecules. 2026 Apr 27;31(9):1449. doi: 10.3390/molecules31091449 (PMC13164609; doi:10.3390/molecules31091449)
Supplement: Supplementary file 1 [file molecules-31-01449-s001.zip › molecules-4205369-supplementary.pdf]

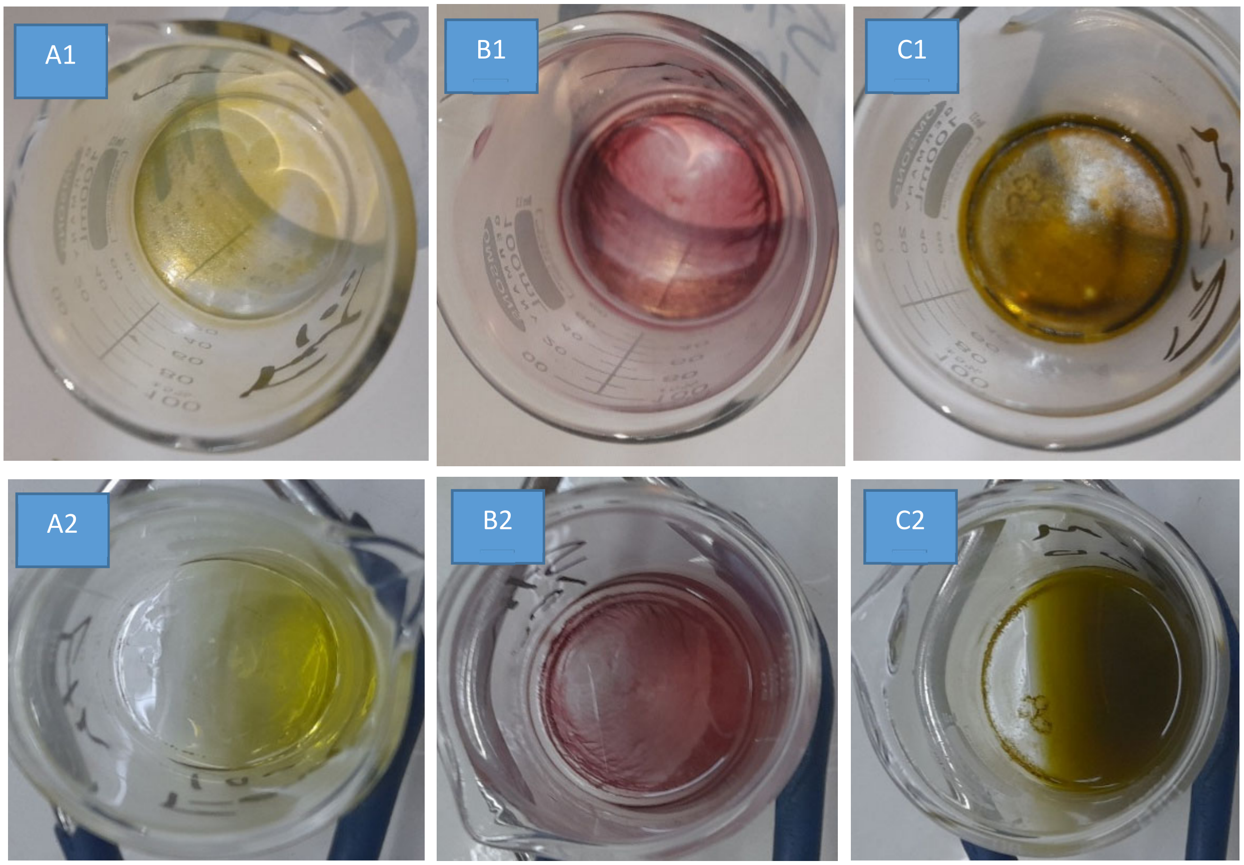

Supplement Figure S1. The thermally treated acetonitrile extracts (10 ml each) of fennel seeds, strawberry, and dry mint were prepared at 100°C for 2 hours and after their redesolving with the same volume of acetonitrile as A1, B1, & C1, and A2, B2, and C2, respectively.

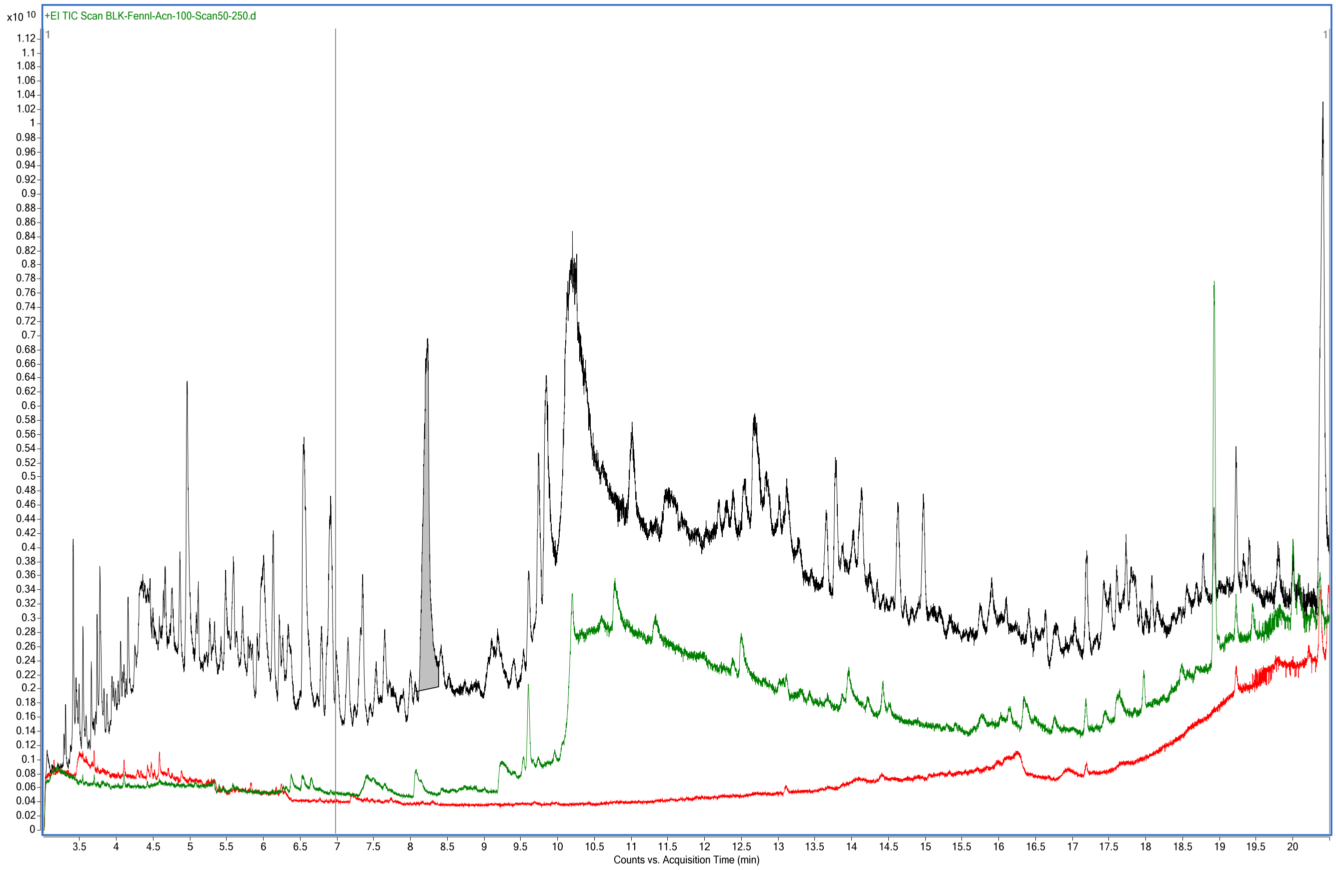

Supplement Figure S2. The total ion chromatogram of the GC-MS scan analysis of acetonitrile extracts of dry mint (black), fennel seed (green), and strawberry (red) after their thermal treatment at 100°C for 2 hours.

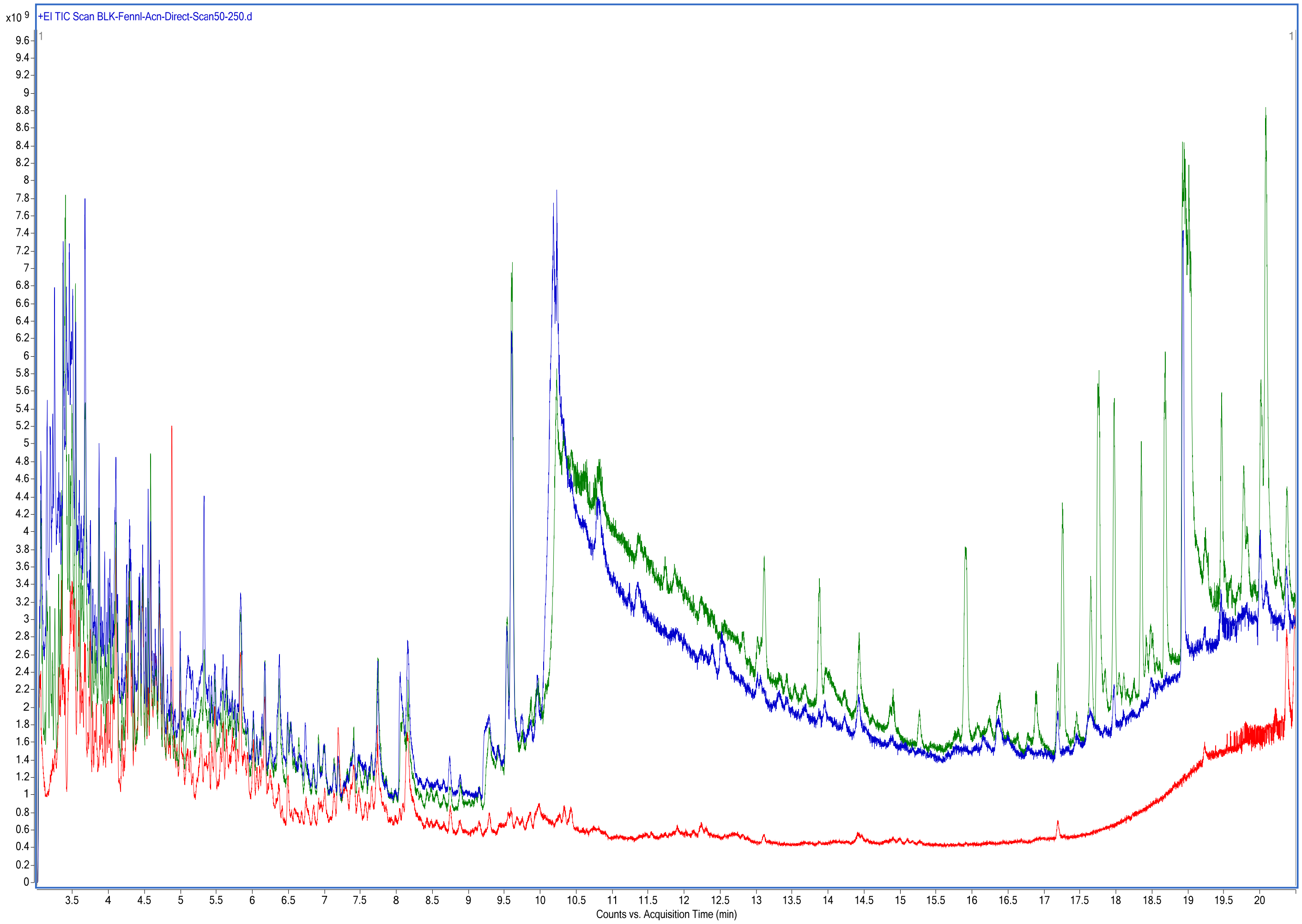

Supplement Figure S3. The total ion chromatograms of the GC-MS scan analysis of acetonitrile and ethyl acetate extracts of fennel seed in blue and green, respectively, with the acetonitrile extract of strawberry in red.

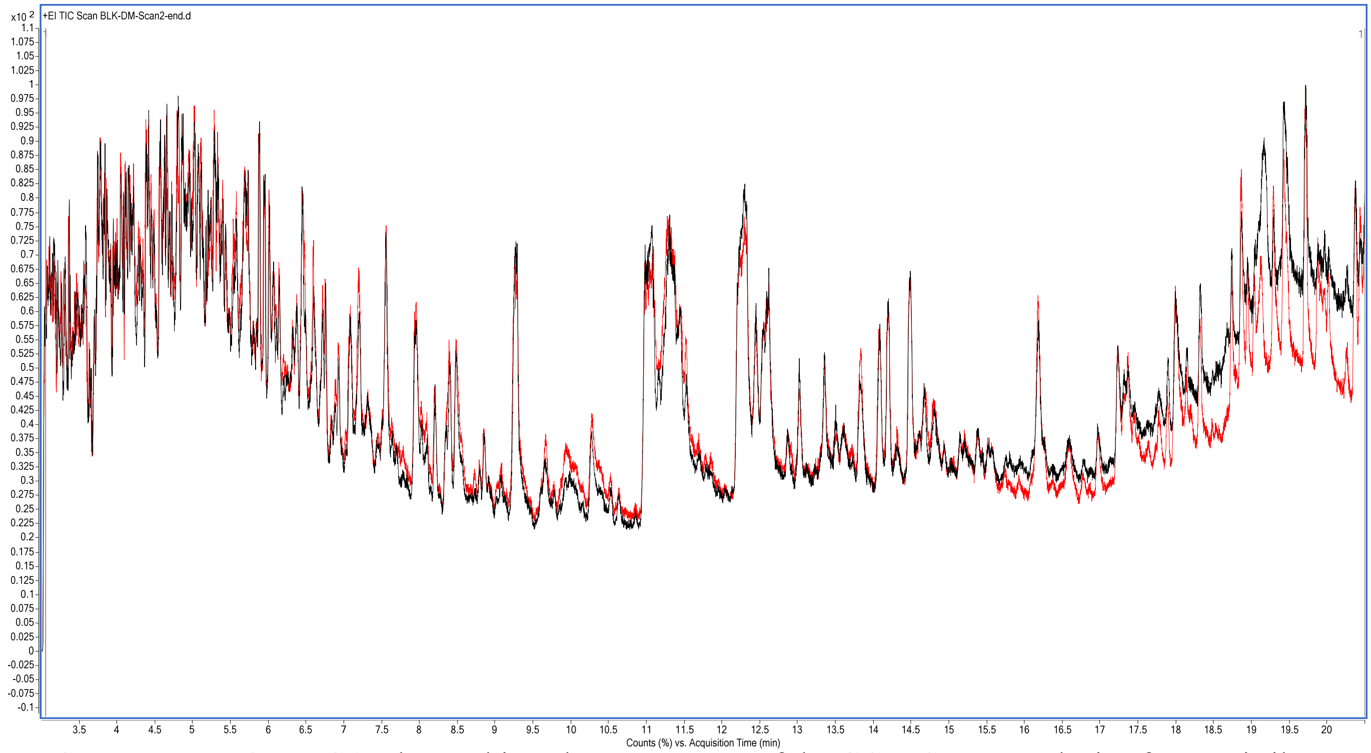

Supplement Figure S4. The total ion chromatograms of the GC-MS scan analysis of acetonitrile **dry mint** extract with back flush cleaning at first batch injection in red and that after repeated injection (n=11) in black.

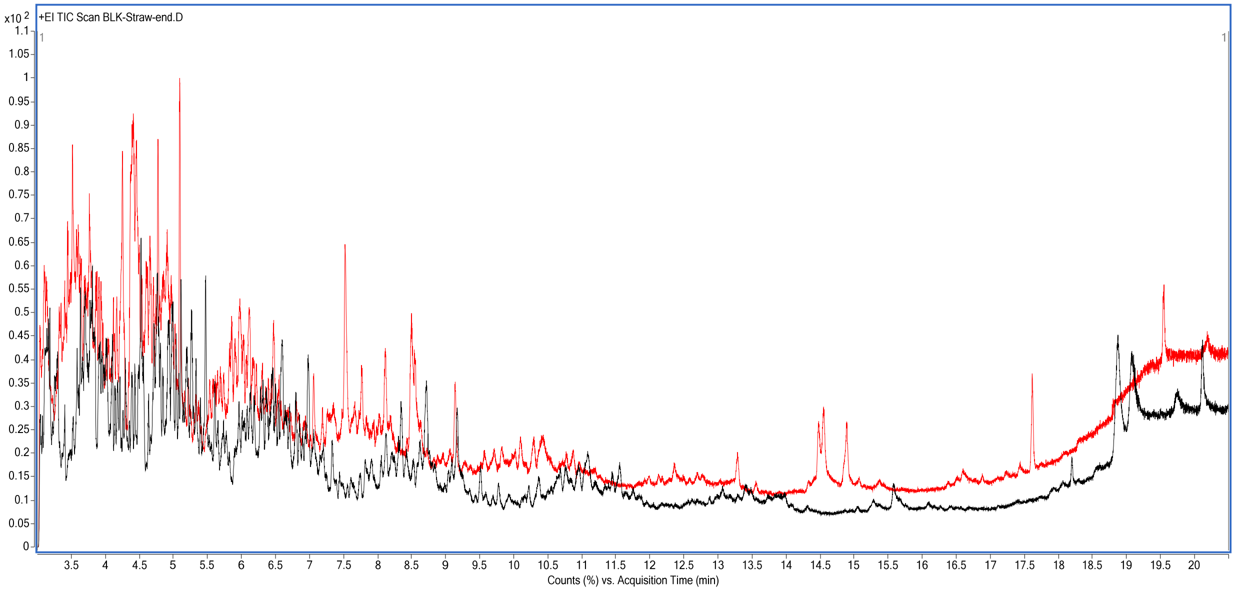

Supplement Figure S5. The total ion chromatograms of the GC-MS scan analysis of acetonitrile strawberry extract (without mixing with fennel as NAP and with applying back flush) at the first batch injection in red and after repeated injection (n=11) in black.

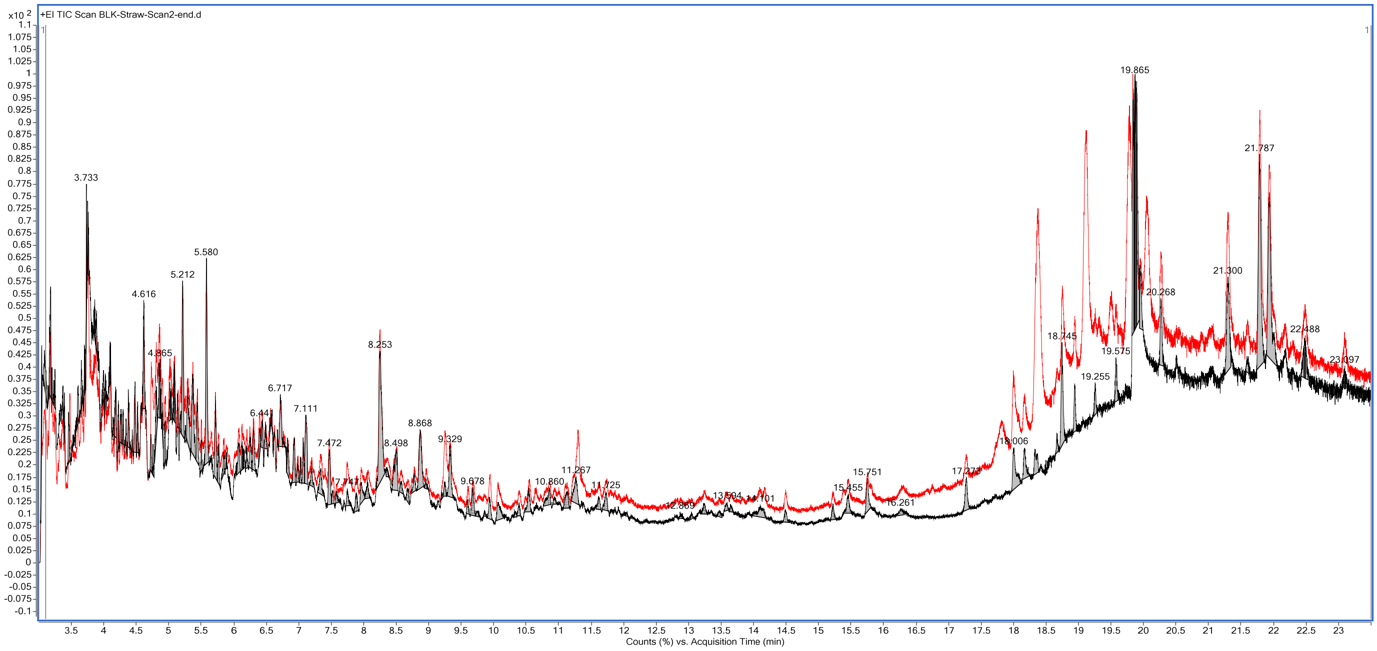

Supplement Figure S6. The total ion chromatograms of the GC-MS scan analysis of acetonitrile strawberry extract (with mixing with fennel as NAP and with applying the same gas flow column cleaning) at first batch injection in red and that after repeated injection (n=11) in black.

***S. Table S1*.** The result of the Intra-day measurements in strawberry and dry mint using the developed methods: Recovery (Rec, %) and relative standard deviation (RSD, %) values for the targeted pesticides at various fortification levels.

Pesticide	Strawberry *				Dry Mint**					
	0.01 mg/kg		0.05 mg/kg		0.01 mg/kg		0.05 mg/kg		0.25 mg/kg	
	Rec	RSD	Rec	RSD	Rec	RSD	Rec	RSD	Rec	RSD
Acrinathrin	96	3	96	6	77	11	71	11	89	4
Alachlor	108	1	95	1	121	9	103	6	110	1
Ametryn	103	3	93	3	<LOQ	<LOQ	75	7	101	2
Atraton	90	15	92	2	<LOQ	<LOQ	62	11	92	9
Atrazine	103	2	92	2	103	4	104	6	89	9
Azinphos-ethyl	87	4	81	3	<LOQ	<LOQ	113	6	77	17
Azinphos-methyl	96	8	67	6	85	8	63	12	100	4
Benalaxyl	104	6	93	5	<LOQ	<LOQ	108	5	110	2
Bifenthrin	111	2	95	2	138	9	91	4	121	2
Bifinazate	83	5	91	3	86	9	94	4	76	7
Biphenyl	108	4	91	2	<LOQ	<LOQ	99	4	103	1
Bitertanol	97	2	98	4	<LOQ	<LOQ	108	6	101	3
Boscalid	98	3	91	2	108	3	106	7	74	9
Bromophos-ethyl	108	1	98	1	78	3	85	3	106	1
Bromophos-methyl	105	3	94	1	90	3	95	4	105	1
Bromopropylate	101	1	95	1	<LOQ	<LOQ	67	4	102	1
Bromuconazole I	104	2	85	3	<LOQ	<LOQ	105	5	94	5
Bromuconazole II	109	4	91	2	89	8	87	7	93	2
Bupirimate	111	1	94	2	115	4	134	6	84	14

Buprofezin	106	2	96	2	113	14	102	6	104	1
Butachlor	111	2	96	1	125	5	135	2	111	5
Butralin	101	3	91	1	80	4	88	5	108	2
Cadusafos	101	4	91	2	101	7	103	5	105	1
Carboxin	88	10	88	4	98	9	88	9	81	13
Chlordane cis-(alpha)	112	2	96	1	95	8	119	3	103	5
Chlordane trans-(gamma)	109	1	97	1	70	4	77	3	100	3
Chlorfenapyr	97	6	100	3	99	7	92	12	110	2
Chlorfenvinphos	99	2	90	1	92	5	96	5	110	1
Chlorobenzilate	104	3	93	3	97	1	99	4	109	1
Chlorpropham	102	3	92	2	92	3	96	6	105	2
Chlorpyrifos	110	2	96	1	114	2	92	4	107	1
Chlorpyrifos-methyl	107	1	94	1	88	3	92	5	106	1
Chlorthal-dimethyl	108	1	95	1	94	1	99	4	107	1
Chlorthiophos	105	3	96	1	82	2	91	5	110	1
Chlozolinate	107	1	95	2	88	5	91	4	111	1
Clodinafop-propargyl ester	96	2	92	3	87	4	90	7	106	3
Coumaphos	86	8	78	6	78	7	78	8	94	2
Cyanophos	107	2	93	1	90	4	91	6	104	1
Cyfluthrin	98	1	96	2	100	7	79	9	121	2
Cyhalothrin lambda-	104	2	95	4	75	11	74	9	115	3
Cypermethrin	104	3	99	2	96	7	78	7	116	2
Cyproconazole	101	3	92	2	98	4	102	5	108	1

Cyprodinil	105	2	93	1	87	2	91	4	104	1
DDD op`-	106	1	96	1	89	2	85	4	108	2
DDE pp`-	106	1	95	1	71	2	75	2	100	1
Deltamethrin	101	2	90	3	<LOQ	<LOQ	<LOQ	<LOQ	95	6
Demeton-s-methyl	106	19	88	12	<LOQ	<LOQ	115	4	98	6
Diazinon	108	3	97	1	98	2	106	4	105	3
Dichlobenil	108	2	90	1	98	3	100	5	104	1
Dichlofenthion	106	1	97	1	90	1	95	4	107	1
Dichlorvos	101	2	89	1	109	6	117	7	75	6
Diclofop methyl	104	3	93	1	87	4	93	5	109	2
Dicloran	95	8	99	6	<LOQ	<LOQ	96	6	99	3
Dieldrin	105	3	95	1	85	6	86	3	102	2
Diethofencarb	98	1	91	1	93	5	99	4	111	1
Difenoconazol I	111	4	102	5	111	7	108	8	72	7
Dimethachlor	124	3	93	1	108	6	107	6	109	2
Dimethoate	60	18	71	13	89	10	84	9	100	2
Diniconazole	93	5	89	2	97	4	105	5	108	1
Diphenylamine	119	3	89	1	<LOQ	<LOQ	91	7	91	3
Disulfoton	105	3	94	1	88	9	88	6	97	5
Disulfoton sulfone	104	6	93	2	<LOQ	<LOQ	105	9	99	3
Ditalimfos	81	9	81	3	94	9	81	7	88	4
Endosulfan alpha-	115	9	94	1	106	7	118	4	107	6
Endosulfan-sulfate	107	2	93	1	69	11	68	9	111	3

Endrin	114	9	95	2	<LOQ	<LOQ	78	11	96	5
Epoxiconazole II	104	2	91	1	94	2	95	6	101	1
Ethion	102	3	94	3	102	4	98	5	117	1
Ethofumesate	109	2	94	1	106	8	117	3	92	6
Ethoprophos	107	1	94	1	99	2	100	4	109	1
Etofenprox	116	1	98	2	87	4	83	4	112	2
Etoxazole	104	3	94	1	<LOQ	<LOQ	105	4	105	6
Etridiazole	108	2	91	1	112	5	89	6	79	4
Fenarimol	102	2	94	2	113	21	107	5	101	3
Fenazaquin	91	3	88	2	<LOQ	<LOQ	97	3	119	1
Fenbuconazole	105	5	95	3	105	9	104	7	77	7
Fenitrothion	90	4	86	2	100	3	109	5	108	3
Fenoxaprop-P-ethyl	104	2	93	6	104	4	108	6	78	11
Fenoxycarb	107	7	78	6	133	13	79	9	76	3
Fenpropathrin	113	4	97	2	82	6	92	5	117	1
Fenpropimorph	108	2	96	1	91	2	94	4	102	1
Fenvalerate	101	1	98	2	<LOQ	<LOQ	101	5	115	2
Fluazifop-p-butyl	107	2	96	1	91	3	98	4	113	2
Flucythrinate	109	2	103	3	<LOQ	<LOQ	100	5	117	1
Fludioxonil	91	3	84	1	83	5	80	9	92	2
Fluquinconazole	99	2	91	2	89	8	92	6	87	7
Flusilazole	101	2	92	1	91	8	97	4	104	2
Fluvalinate tau-	106	2	99	3	76	8	70	10	119	3

Formothion	110	2	93	1	108	4	104	6	98	1
HCH alpha-	110	1	93	1	93	2	97	3	103	1
HCH beta-	119	2	96	2	110	2	98	5	105	1
HCH delta	104	3	92	1	<LOQ	<LOQ	89	5	103	2
HCH gamma-	105	3	91	0	99	2	95	4	100	1
Heptachlor	110	1	94	1	71	2	75	3	91	1
Heptachlor-endo-epoxide (trans-)	105	1	94	2	<LOQ	<LOQ	101	3	106	1
Heptachlor-exo-epoxide (cis-)	113	3	96	1	82	5	91	4	104	1
Heptenophos	103	2	91	1	94	8	89	9	106	2
Hexachlorobenzene (HCB)	101	1	92	1	64	3	65	2	83	2
Hexaconazole	98	2	92	2	105	6	110	3	109	1
Hexazinone	140	5	86	6	105	6	98	8	75	9
Imazalil	109	7	100	3	100	12	88	6	82	4
Iprobenfos	101	2	93	1	100	1	100	5	110	1
Iprodione	102	2	88	2	82	8	82	9	106	2
Isofenphos	105	1	95	1	<LOQ	<LOQ	100	8	106	3
Isofenphos-methyl	110	1	96	1	99	2	104	4	112	1
Isoprothiolane	102	2	93	2	97	3	104	4	111	1
Kresoxim-methyl	102	1	95	1	116	7	104	5	107	3
Linuron	105	2	96	1	96	3	104	5	106	0
Malathion	101	2	92	1	95	3	96	6	112	2
Mecarbam	98	7	91	3	100	7	101	9	108	4
Mefenacet	76	6	72	3	95	8	82	9	81	3

Mefenpyr-diethyl	108	2	94	2	93	4	101	5	112	1
Mepronil	100	5	89	3	<LOQ	<LOQ	72	8	111	3
Metalaxyl	118	4	90	2	<LOQ	<LOQ	106	9	116	1
Metazachlor	105	2	94	1	121	6	97	10	113	2
Methacrifos	110	1	93	1	103	3	104	6	97	1
Methidathion	104	3	88	3	97	6	90	3	109	2
Metribuzin	103	5	92	1	92	5	95	4	106	5
Mirex	108	1	94	0	<LOQ	<LOQ	<LOQ	<LOQ	84	2
Monolinuron	84	13	84	12	105	8	124	7	79	13
Myclobutanil	104	2	93	2	99	4	99	5	110	1
Napropamide	117	11	97	2	131	5	80	6	105	1
Nuarimol	102	2	91	1	100	3	106	5	108	1
Oxadiaxyl	94	5	92	4	94	5	91	6	100	3
Oxadiazon	109	1	97	1	91	1	97	3	109	1
Oxyfluorfen	96	2	90	3	85	9	90	6	110	2
Paraoxon-ethyl	78	11	76	6	68	16	61	11	101	6
Parathion-ethyl	95	3	88	1	87	4	92	5	108	1
Parathion-methyl	92	4	87	1	86	5	82	7	103	2
PCB 028	99	2	98	2	<LOQ	<LOQ	61	3	80	4
PCB 052	102	1	98	2	66	5	75	2	81	3
PCB 101	106	2	99	1	<LOQ	<LOQ	<LOQ	<LOQ	76	3
PCB 118	99	1	98	2	<LOQ	<LOQ	<LOQ	<LOQ	74	4
PCB 138	104	2	99	1	<LOQ	<LOQ	<LOQ	<LOQ	75	3

PCB 153	102	2	100	1	<LOQ	<LOQ	<LOQ	<LOQ	73	3
PCB 180	93	1	97	2	<LOQ	<LOQ	<LOQ	<LOQ	73	3
Penconazole	107	1	93	1	89	6	99	6	110	2
Pendimethalin	102	3	92	1	101	4	92	4	104	1
Pentachloroanisole	105	3	94	3	70	11	75	9	88	7
Pentachlorobenzene	99	2	91	1	74	2	77	3	86	1
Permethrin	93	3	98	2	<LOQ	<LOQ	110	5	80	3
Phenthoate	106	0	96	1	96	3	101	4	112	2
Phenylphenol ortho-(2-phenylphenol)(OPP)	104	1	88	1	94	4	94	6	104	2
Phorate	105	2	95	1	97	5	102	4	105	1
Phosalone	99	1	86	3	<LOQ	<LOQ	76	7	109	2
Phosmet	84	9	69	6	67	12	62	12	100	3
Piperonyl-butoxide	105	2	95	2	117	1	104	5	104	5
Pirimicarb	104	2	93	1	96	3	98	5	105	2
Pirimiphos-ethyl	107	2	96	0	91	4	101	4	110	1
Pirimiphos-methyl	103	1	95	1	110	4	131	3	112	5
Procymidone	110	2	95	1	93	3	97	5	108	1
Profenofos	98	2	88	1	81	6	81	6	104	2
Profluralin	102	2	94	2	80	5	89	4	106	2
Prometon	108	2	95	2	<LOQ	<LOQ	118	4	113	1
Prometryn	117	2	94	1	89	7	97	4	108	1
Propanil	94	13	78	11	88	5	89	6	101	4
Propazine	106	2	95	1	116	6	108	5	94	7

Propiconazol	101	3	95	2	111	4	93	8	110	1
Prosulfocarb	106	3	94	1	96	6	90	14	103	3
Prothiofos	108	2	96	1	77	6	85	2	105	1
Pyrazofos	99	2	92	4	110	3	93	7	109	2
Pyridaben	91	3	92	2	<LOQ	<LOQ	97	5	112	1
Pyridaphenthion	93	4	84	3	85	10	85	8	104	2
Pyrifenox I	104	5	94	9	79	7	87	6	96	2
Pyrifenox II	100	5	91	5	81	5	81	7	94	1
Pyrimethanil	107	1	94	1	93	3	95	5	103	1
Pyriproxyfen	102	3	91	1	89	4	88	6	105	2
Quinalphos	105	3	94	1	102	4	99	5	106	1
Quintozene	112	3	90	1	73	4	79	3	91	2
Simazine	93	12	110	2	<LOQ	<LOQ	119	3	89	8
Spiromesifen	106	7	92	3	102	11	92	6	106	7
Spiroxamine	111	20	94	7	122	9	80	6	104	3
Sulfotep	112	1	97	1	97	3	105	4	107	1
Tebuconazole	104	2	92	2	93	4	95	6	106	1
Tebufenpyrad	97	1	93	2	89	3	96	4	109	1
Tecnazene	105	2	92	1	139	2	96	4	99	1
Tefluthrin	108	1	98	1	91	1	92	3	115	1
Terbufos	113	2	98	1	93	1	100	4	109	1
Terbuthylazine	107	3	94	1	101	6	114	4	92	8
Terbutryn	107	3	93	1	<LOQ	<LOQ	<LOQ	<LOQ	103	8

Tetraconazole	104	2	93	2	94	5	103	4	113	1
Tetradifon	100	2	93	1	<LOQ	<LOQ	99	4	102	1
Tetramethrin	82	1	87	2	<LOQ	<LOQ	96	14	93	5
Thiobencarb	110	9	93	3	97	8	93	5	105	2
Thiometon	84	18	86	2	<LOQ	<LOQ	103	10	99	10
Tolclofos-methyl	111	1	96	1	92	2	97	4	107	1
Tolylfluanid	76	1	78	2	<LOQ	<LOQ	<LOQ	<LOQ	96	14
Triadimefon	104	2	94	1	100	5	104	4	113	1
Triadimenol	111	7	98	3	<LOQ	<LOQ	104	4	113	2
Triazophos	97	2	88	2	93	6	87	8	107	2
Trifloxystrobin	99	1	95	2	137	4	105	4	112	2
Triflumizole	106	4	94	1	117	9	113	5	85	6
Trifluralin	130	1	93	1	78	3	86	6	102	1
Triticonazole	96	2	88	3	111	3	116	6	88	10
Vinclozolin	106	2	95	2	98	4	107	6	109	1

*Mixing with concentrated fennel EtOAc extract as NAP and applying back flush column clean up.

** Applying fivefold sample dilution, using column cleaning by same forward carrier gas flow

S. Table S2. Linearity (Correlation Coefficient, R^2) and Matrix Effect (ME, %) for the targeted pesticides in strawberry and dry mint extracts.

Pesticide	R^2		Me	
	Straw	DM	Straw	DM
Phosmet	0.93	0.95	-99.33	-99.35
Dicloran	0.93	0.95	-99.24	-99.20
Paraoxon-ethyl	0.93	0.95	-99.09	-99.21
Simazine	0.94	0.95	-99.14	-98.96
Azinphos-ethyl	0.94	0.95	-99.19	-99.15
Azinphos-methyl	0.94	0.95	-99.27	-99.45
Pyridaphenthion	0.94	0.95	-99.16	-99.41
Propanil	0.94	0.95	-99.05	-99.23
Imazalil	0.94	0.96	-99.08	-99.14
Coumaphos	0.94	0.95	-99.38	-99.55
Pyrazofos	0.94	0.95	-99.20	-99.26
Monolinuron	0.94	0.95	-99.09	-98.83
Butralin	0.94	0.95	-99.17	-99.18
Acrinathrin	0.94	0.95	-99.15	-99.35
Pendimethalin	0.94	0.95	-99.18	-99.16
Oxyfluorfen	0.94	0.95	-99.16	-99.25
Clodinafop-propargyl ester	0.94	0.95	-99.10	-99.29
Bromuconazole II	0.94	0.95	-99.34	-99.21
Tetramethrin	0.94	0.95	-99.07	-99.17
Parathion-methyl	0.94	0.95	-99.22	-99.21
Mecarbam	0.94	0.95	-99.28	-99.18
Difenoconazol I	0.94	0.96	-99.11	-99.30
Parathion-ethyl	0.94	0.95	-99.16	-99.17
Fenitrothion	0.94	0.95	-99.25	-98.99
Fenpropimorph	0.94	0.96	-99.92	-99.89
Fenoxaprop-P-ethyl	0.94	0.95	-99.22	-99.23
Bifinazate	0.94	0.96	-99.17	-99.17
Hexazinone	0.94	0.96	-98.38	-99.18
Oxadiaxyl	0.94	0.95	-99.33	-99.16
Carboxin	0.94	0.95	-99.29	-99.14
Mefenacet	0.94	0.96	-99.21	-99.35
Phosalone	0.94	0.95	-99.22	-99.28
Nuarimol	0.94	0.95	-99.22	-99.11
Fenoxycarb	0.94	0.95	-99.50	-98.05

Triticonazole	0.95	0.96	-99.11	-99.18
Atraton	0.95	0.95	-99.30	-99.06
Dimethoate	0.95	0.96	-99.41	-99.47
Mefenpyr-diethyl	0.95	0.95	-99.18	-99.14
Tebuconazole	0.95	0.95	-99.16	-99.14
Fenbuconazole	0.95	0.96	-99.16	-99.02
Cyhalothrin lambda-	0.95	0.95	-99.20	-99.11
Piperonyl-butoxide	0.95	0.96	-99.07	-99.09
Endrin	0.95	0.95	-99.41	-99.22
Spiromesifen	0.95	0.95	-99.30	-99.27
Fluquinconazole	0.95	0.95	-99.25	-99.14
Ethion	0.95	0.95	-99.11	-99.28
Profluralin	0.95	0.95	-99.23	-99.12
Flusilazole	0.95	0.95	-99.29	-99.15
Bromopropylate	0.95	0.96	-99.26	-99.17
Bupirimate	0.95	0.95	-99.22	-99.09
Etofenprox	0.95	0.95	-99.18	-99.09
Iprodione	0.95	0.95	-99.36	-99.40
Fenvalerate	0.95	0.95	-99.21	-99.06
Fenpropathrin	0.95	0.95	-99.21	-99.13
Chlorfenapyr	0.95	0.95	-71.65	-72.96
Fenarimol	0.95	0.95	-99.14	-99.11
Epoxiconazole II	0.95	0.95	-99.26	-99.20
Myclobutanil	0.95	0.95	-99.22	-99.03
Etoxazole	0.95	0.95	-99.04	-99.08
Fluazifop-p-butyl	0.95	0.95	-99.15	-99.24
Fludioxonil	0.95	0.96	-99.24	-99.21
Triazophos	0.95	0.95	-99.23	-99.24
Quintozene	0.95	0.95	-99.23	-99.12
Fluvalinate tau-	0.95	0.95	-99.20	-99.36
Ametryn	0.95	0.95	-99.23	-99.06
Endosulfan-sulfate	0.95	0.94	-99.32	-99.25
Thiobencarb	0.95	0.95	-99.15	-99.15
PCB 153	0.95	0.96	-99.17	-99.25
Permethrin	0.95	0.95	-99.12	-99.02
Buprofezin	0.95	0.95	-97.25	-99.05
Pyrimethanil	0.95	0.96	-99.20	-99.04
Chlorfenvinphos	0.95	0.95	-99.21	-99.20
Tetradifon	0.95	0.95	-99.22	-99.07
Kresoxim-methyl	0.95	0.95	-99.24	-99.04
Cyfluthrin	0.95	0.95	-99.12	-99.09

Flucythrinate	0.95	0.95	-99.13	-99.13
Triadimenol	0.95	0.96	-99.13	-99.14
Metribuzin	0.95	0.95	-95.61	-99.03
Metazachlor	0.95	0.95	-99.22	-99.12
Cyproconazole	0.95	0.95	-99.15	-99.13
Boscalid	0.95	0.97	-99.13	-98.89
Diniconazole	0.95	0.95	-99.07	-99.31
Trifluralin	0.95	0.95	-98.94	-98.93
Hexaconazole	0.95	0.95	-99.18	-99.17
Propiconazol	0.95	0.95	-99.19	-99.11
Triadimefon	0.95	0.95	-98.90	-98.69
Diclofop methyl	0.95	0.95	-99.20	-99.13
Pyrifenox II	0.95	0.95	-99.25	-99.20
Diethofencarb	0.95	0.95	-99.14	-99.22
Heptenophos	0.95	0.95	-99.58	-99.47
Chlorpyrifos-methyl	0.95	0.95	-99.28	-99.22
Penconazole	0.95	0.95	-99.22	-99.09
Vinclozolin	0.95	0.95	-99.33	-99.17
Iprobenfos	0.95	0.96	-99.17	-99.13
PCB 138	0.95	0.95	-99.24	-99.24
Isoprothiolane	0.95	0.95	-99.25	-99.19
Linuron	0.95	0.95	-99.37	-99.10
Butachlor	0.95	0.94	-99.23	-99.11
Bromophos-methyl	0.95	0.95	-99.33	-99.27
Metalaxyl	0.95	0.95	-99.36	-99.25
PCB 028	0.95	0.95	-99.20	-99.18
Pirimiphos-ethyl	0.95	0.95	-99.26	-99.23
Atrazine	0.95	0.95	-99.20	-99.08
Cypermethrin	0.95	0.95	-99.20	-99.09
Prometryn	0.95	0.95	-99.19	-99.19
Propazine	0.95	0.95	-99.27	-99.06
Chlorpyrifos	0.95	0.95	-99.28	-99.21
PCB 118	0.95	0.95	-99.17	-99.20
Chlorthiophos	0.95	0.95	-99.28	-99.26
Bitertanol	0.95	0.96	-99.14	-99.26
Cyanophos	0.95	0.95	-99.26	-99.13
Chlozolinate	0.95	0.95	-99.27	-99.18
Bromuconazole I	0.95	0.95	-99.11	-99.14
PCB 180	0.95	0.95	-99.32	-99.28

Terbutryn	0.95	0.95	-96.88	-99.05
PCB 052	0.95	0.95	-99.17	-99.07
Pyriproxyfen	0.95	0.95	-99.07	-99.09
Profenofos	0.95	0.95	-99.22	-99.24
Diphenylamine	0.95	0.95	-99.35	-99.16
Deltamethrin	0.95	0.94	-99.10	-99.06
Tebufenpyrad	0.95	0.95	-99.29	-99.18
Malathion	0.95	0.95	-99.24	-99.11
Cyprodinil	0.95	0.95	-99.17	-99.12
Bromophos-ethyl	0.95	0.95	-99.32	-99.26
Diazinon	0.95	0.95	-99.31	-99.17
Quinalphos	0.95	0.96	-99.20	-99.03
PCB 101	0.95	0.95	-99.21	-99.19
Phenthoate	0.95	0.95	-99.20	-99.16
HCH delta	0.95	0.95	-99.38	-99.13
HCH gamma-	0.95	0.95	-99.50	-99.11
Prothiofos	0.95	0.95	-99.20	-99.25
Terbuthylazine	0.95	0.96	-99.27	-98.97
Prometon	0.95	0.95	-99.29	-99.16
Tecnazene	0.95	0.95	-99.34	-99.12
Pirimiphos-methyl	0.95	0.95	-99.30	-99.06
Napropamide	0.95	0.95	-99.12	-99.09
Pirimicarb	0.95	0.95	-99.31	-99.10
Alachlor	0.95	0.95	-99.26	-99.07
Etridiazole	0.95	0.95	-99.63	-99.46
Trifloxystrobin	0.95	0.96	-99.22	-99.04
Pentachlorobenzene	0.95	0.95	-99.54	-99.40
Chlorpropham	0.95	0.95	-99.31	-99.16
Tolylfluanid	0.95	0.94	-98.97	-98.59
Ethofumesate	0.95	0.95	-99.27	-99.17
Procymidone	0.95	0.95	-99.24	-99.15
Benalaxyl	0.95	0.95	-99.18	-99.10
Chlordane cis- (alpha)	0.95	0.94	-99.34	-99.26
Chlorthal-dimethyl	0.95	0.95	-99.28	-99.21
Spiroxamine	0.95	0.95	-99.02	-99.02
Pentachloroanisole	0.95	0.95	-99.38	-99.30
Dieldrin	0.95	0.95	-99.36	-99.26
Chlordane trans- (gamma)	0.95	0.95	-99.37	-99.25
Triflumizole	0.95	0.94	-99.25	-99.09
Isofenphos	0.95	0.95	-99.96	-98.90
Disulfoton sulfone	0.95	0.95	-99.25	-99.08

Heptachlor-exo-epoxide (cis-)	0.95	0.95	-99.35	-99.24
Cadusafos	0.95	0.95	-99.21	-99.06
Sulfotep	0.95	0.95	-99.28	-99.15
Heptachlor	0.95	0.95	-99.43	-99.26
Phorate	0.95	0.95	-99.26	-99.08
Ethoprophos	0.95	0.95	-99.30	-99.06
Tolclofos-methyl	0.95	0.95	-99.26	-99.16
Tetraconazole	0.95	0.95	-99.28	-99.28
DDD op`-	0.95	0.95	-98.55	-98.37
Disulfoton	0.95	0.95	-99.60	-98.94
Mirex	0.95	0.95	-99.34	-99.26
Heptachlor-endo-epoxide (trans-)	0.95	0.95	-99.25	-99.03
Demeton-s-methyl	0.95	0.95	-99.34	-99.08
Endosulfan alpha-	0.95	0.95	-99.32	-99.08
Hexachlorobenzene (HCB)	0.95	0.95	-99.28	-99.10
Prosulfocarb	0.95	0.96	-99.16	-98.99
Oxadiazon	0.95	0.95	-99.31	-99.16
Terbufos	0.95	0.96	-99.20	-98.97
Biphenyl	0.95	0.95	-99.48	-99.26
Thiometon	0.95	0.95	-99.34	-99.08
Ditalimfos	0.95	0.96	-99.21	-99.13
HCH alpha-	0.95	0.95	-99.51	-99.04
Methidathion	0.95	0.95	-97.69	-99.98
Dichlofenthion	0.95	0.95	-99.28	-99.19
HCH beta-	0.95	0.95	-99.41	-99.04
Dichlorvos	0.95	0.94	-99.50	-99.00
Formothion	0.95	0.95	-99.55	-99.36
DDE pp`-	0.95	0.96	-99.29	-99.17
Isofenphos-methyl	0.95	0.96	-99.15	-99.05
Fenazaquin	0.95	0.96	-99.19	-98.98
Mepronil	0.96	0.98	-99.12	-98.80
Methacrifos	0.96	0.95	-99.50	-99.31
Phenylphenol ortho-(2-phenylphenol)(OPP)	0.96	0.96	-99.61	-99.42
Dichlobenil	0.96	0.97	-99.48	-99.06
Pyrifenox I	0.96	0.95	-99.18	-99.19
Dimethachlor	0.96	0.96	-99.19	-99.01
Pyridaben	0.96	0.96	-99.12	-98.96
Chlorobenzilate	0.96	0.97	-99.22	-99.05
Tefluthrin	0.96	0.96	-99.16	-99.01

Bifenthrin	0.97	0.97	-99.10	-98.99

S. Table S3. The mass transitions (precursor and product ions), collision energies (CE), and retention times (RT) for the targeted pesticides

Pesticide	Precurser Ion	Daughter Ion	CE	RT(min)
Acrinathrin	289	93	5	16.72
Acrinathrin	209	141	20	16.72
Acrinathrin	208	181	5	16.72
Alachlor	188.1	160.1	10	8.88
Alachlor	160.05	130	30	8.88
Aldrin	293	186	30	9.69
Aldrin	263	193	25	9.69
Aldrin	262.9	190.9	40	9.69
Ametryn	227.15	170.1	30	8.89
Ametryn	227.15	152.1	20	8.89
Atraton	211	196	15	7.03
Atraton	211	169	15	7.03
Atrazine	200.1	122.1	10	7.24
Atrazine	200.1	103.9	20	7.24
Atrazine	200.1	94.1	20	7.24
Azinphos-ethyl	160	132	0	16.85
Azinphos-ethyl	160	104	8	16.85
Azinphos-ethyl	132	104	4	16.85
Azinphos-ethyl	132	77	12	16.85
Azinphos-methyl	160.05	132.1	5	15.05
Azinphos-methyl	160.05	102.1	15	15.05
Azinphos-methyl	160.05	77.1	20	15.05
Benalaxyl	204	176	5	13.64
Benalaxyl	148	105	17	13.64
Bifenthrin	181.1	166.1	15	15.13
Bifenthrin	181	165	25	15.13
Bifinazate	300	258	15	15.19
Bifinazate	258	199	15	15.19
Biphenyl	154	152	20	4.60
Biphenyl	154	126	40	4.60
Biphenyl	154	102	20	4.60
Bitertanol	170	141	20	17.32
Bitertanol	170	115	35	17.32
Boscalid	342	140	10	18.44

Boscalid	140	112	10	18.44
Bromophos-ethyl	358.9	302.9	15	11.18
Bromophos-ethyl	358.7	331	5	11.18
Bromophos-methyl	330.9	315.9	20	10.20
Bromophos-methyl	330.9	285.9	35	10.20
Bromopropylate	341	185	20	15.06
Bromopropylate	183	155	15	15.06
Bromuconazole I	294.8	173	15	14.98
Bromuconazole I	172.9	145	15	14.98
Bupirimate	316.1	208.2	5	12.27
Bupirimate	273	193	5	12.27
Bupirimate	273	108	15	12.27
Buprofezin	172	57	15	12.19
Buprofezin	105	104	15	12.19
Butachlor	236.8	188.3	25	11.44
Butachlor	236.8	160.3	25	11.44
Butralin	265.9	220.2	10	10.14
Butralin	265.9	190.2	10	10.14
Butralin	265.9	174.1	10	10.14
Cadusafos	213	73	10	6.67
Cadusafos	159	131	10	6.67
Carboxin	235	162.1	5	12.17
Carboxin	235	143	5	12.17
Carboxin	144	87	5	12.17
Chlordane Cis, trans-	372.9	265.9	30	11.12, 11.32
Chlordane Cis, trans-	372.9	263.9	25	11.12, 11.33
Chlorfenapyr	408	59	10	12.57
Chlorfenapyr	59	31	10	12.57
Chlorfenapyr	59	29	10	12.57
Chlorobenzilate	251	139	12	12.72
Chlorobenzilate	139	111	15	12.72
Chlorobenzilate	139	75	15	12.72
Chlorpropham	213	171	5	6.33
Chlorpropham	213	127	5	6.33
Chlorpyrifos	313.8	258	14	9.78
Chlorpyrifos	196.9	168.9	15	9.78
Chlorpyrifos	196.9	107	40	9.78
Chlorpyrifos-methyl	286	270.9	20	8.73

Chlorpyrifos-methyl	286	93	20	8.73
Chlorthiophos	324.9	269.2	15	13.15
Chlorthiophos	268.7	205.1	15	13.15
Chlozolinate	188.1	147.1	15	10.61
Chlozolinate	186	145	15	10.61
Clodinafop-propargyl ester	349	266.1	10	13.87
Clodinafop-propargyl ester	349	238.1	15	13.87
Clodinafop-propargyl ester	266	222.1	20	13.87
Coumaphos	362	81	30	17.70
Coumaphos	361.9	226.2	15	17.70
Coumaphos	361.9	109.2	15	17.70
Cyanophos	243	116	5	7.55
Cyanophos	243	109	10	7.55
Cyanophos	243	79	30	7.55
Cyfluthrin	226.9	77.1	30	18.16
Cyfluthrin	163	127.1	5	18.16
Cyfluthrin	163	91.1	15	18.16
Cyhalothrin-lambda	197	161	10	16.47
Cyhalothrin-lambda	181.1	152.1	30	16.47
Cyhalothrin-lambda	181.1	127.1	35	16.47
Cypermethrin	181.1	152.1	25	18.47
Cypermethrin	181.1	127.1	35	18.47
Cypermethrin	162.9	127	5	18.47
Cyproconazole	222	125	18	12.52
Cyproconazole	222	82	8	12.52
Cyprodinil	224	208	20	10.38
Cyprodinil	225	224	10	10.38
DDD op`-	237	165	20	12.97
DDD op`-	235	200	8	12.97
DDD op`-	235	199	15	12.97
DDE pp`-	248	176	30	11.96
DDE pp`-	246	211	20	11.96
DDE pp`-	246	176	30	11.96
Deltamethrin	253	172	10	19.84
Deltamethrin	253	93	20	19.84
Deltamethrin	181	152	25	19.84
Demeton-S- methyl	142	79	10	6.14
Demeton-S- methyl	88	60	7	6.14

Diazinon	304	179	15	7.70
Diazinon	179.1	137.2	20	7.70
Dichlobenil	171	136	15	4.44
Dichlobenil	171	100	25	4.44
Dichlofenthion	279	223	10	8.51
Dichlofenthion	279	205	25	8.51
Dichlorvos	185	109	15	3.91
Dichlorvos	185	93	15	3.91
Dichlorvos	109	79	5	3.91
Diclofop methyl	340	253	15	14.24
Diclofop methyl	253	162	15	14.24
Dicloran	205.9	175.9	5	7.17
Dicloran	205.9	123.9	25	7.17
Dieldrin	262.8	193	30	12.02
Dieldrin	262.8	191	30	12.02
Diethofencarb	267	225	5	9.60
Diethofencarb	196	168	5	9.60
Difenoconazol	325	267	16	19.62
Difenoconazol	323	265	15	19.62
Difenoconazol	267	204	16	19.62
Difenoconazol	265	202	20	19.62
Dimethachlor	134.1	105.1	15	8.55
Dimethachlor	134.1	77.1	30	8.55
Dimethoate	229	87	10	7.14
Dimethoate	143	111	10	7.14
Dimethoate	125	47	20	7.14
Diniconazole	270	234	15	12.90
Diniconazole	270	232	10	12.90
Diniconazole	268	332	15	12.90
Diniconazole	268	136	10	12.90
Diphenylamine	169	167	10	6.16
Diphenylamine	169	77	35	6.16
Disulfoton	88.1	60	25	6.96
Disulfoton	88.1	59	25	6.96
Disulfoton sulfone	213	125	7	7.85
Disulfoton sulfone	186	97.1	15	7.85
Disulfoton sulfone	186	81	20	7.85
Ditalimfos	148	102	25	11.54

Ditalimfos	130	102.1	15	11.54
Endosulfan alpha-	241	206	15	11.42
Endosulfan alpha-	239	204	15	11.42
Endosulfan alpha-	195	159	5	11.42
Endosulfan-sulfate	387	253	5	13.79
Endosulfan-sulfate	271.9	236.9	20	13.79
Endosulfan-sulfate	271.9	116.9	40	13.79
Endrin	281	245	30	12.55
Endrin	263	193	30	12.55
Endrin	262.9	190.9	35	12.55
Epoxiconazole II	191.9	138.1	10	14.61
Epoxiconazole II	191.9	111.1	35	14.61
Ethion	231	185	5	13.07
Ethion	231	175	5	13.07
Ethion	231	129	25	13.07
Ethofumesate	285.9	207.3	5	9.36
Ethofumesate	285.9	161.1	15	9.36
Ethoprophos	158	114	5	6.22
Ethoprophos	158	97	15	6.22
Etofenprox	376	163	10	18.64
Etofenprox	163	135	10	18.64
Etofenprox	163	107	15	18.64
Etoxazole	300	270	20	15.34
Etoxazole	204	176	8	15.34
Etridiazole	210.9	182.9	5	5.28
Etridiazole	182.9	139.9	15	5.28
Fenarimol	219	107	10	16.64
Fenarimol	139	111	15	16.64
Fenarimol	139	75	35	16.64
Fenazaquin	160	145.2	5	15.47
Fenazaquin	160	117	20	15.47
Fenbuconazole	198	129	5	18.09
Fenbuconazole	129	102	10	18.09
Fenitrothion	277.1	109	20	9.32
Fenitrothion	276.8	260	5	9.32
Fenoxaprop-P-ethyl	360.8	288.1	9	17.07
Fenoxaprop-P-ethyl	287.8	119.1	9	17.07
Fenoxycarb	255	186	10	15.09

Fenoxycarb	186	109	15	15.09
Fenpropathrin	265	210	15	15.29
Fenpropathrin	209	116	22	15.29
Fenpropimorph	128	110	10	9.73
Fenpropimorph	128	70	15	9.73
Fenvalerate	225	119	15	19.19
Fenvalerate	167.1	89.1	35	19.19
Fenvalerate	167	125	10	19.19
Fluazifop-p-butyl	282	238	15	12.53
Fluazifop-p-butyl	282	91	15	12.53
Flucythrinate	199.1	157.1	10	18.75
Flucythrinate	199.1	107.1	30	18.75
Fludioxonil	248	182	15	11.99
Fludioxonil	248	154	15	11.99
Fludioxonil	248	127	30	11.99
Fluquinconazole	339.9	313.2	15	17.66
Fluquinconazole	339.9	298.2	20	17.66
Flusilazole	233	165	20	12.21
Flusilazole	233	152	20	12.21
Fluvalinate-Tau	250.1	200.1	20	19.34
Fluvalinate-Tau	250	55.2	20	19.34
Fluvalinate-Tau	208.9	141.1	15	19.34
Fluvalinate-Tau	208.9	77	25	19.34
Formothion I	125.1	79	5	5.18
Formothion I	125.1	47	15	5.18
HCH alpha-	219	183	10	6.90
HCH alpha-	181	145	15	6.90
HCH alpha-	181	109	25	6.90
HCH beta, gama	219	183	10	7.41, 7.49
HCH beta, gama	181	145	15	7.41, 7.49
HCH beta, gama	181	109	30	7.41, 7.49
HCH delta	181	109	30	7.87
HCH delta	219	183	10	7.87
HCH delta	181	145	15	7.87
Heptachlor	274	239	20	8.94
Heptachlor	271.9	236.8	25	8.94
Heptachlor	271.9	116.9	25	8.94
Heptachlor exo-epoxide isomer B	353	282	15	10.58

Heptachlor exo-epoxide isomer B	353	263	15	10.58
Heptachlor-endo- Epoxide (trans-)	183	155	25	10.68
Heptachlor-endo- Epoxide (trans-)	183	118.9	25	10.68
Heptenophos	250	124	25	5.77
Heptenophos	250	89	15	5.77
Heptenophos	215	200	15	5.77
Hexachlorobenzene (HCB)	283.9	248.8	25	7.07
Hexachlorobenzene (HCB)	283.9	213.9	35	7.07
Hexaconazole	214	159	22	11.73
Hexaconazole	213.9	172	22	11.73
Hexazinone	171.1	85.1	15	14.12
Hexazinone	171.1	71.1	15	14.12
Imazalil	217	175	4	11.85
Imazalil	214.9	173	4	11.85
Imazalil	173	109	25	11.85
Iprobenfos	204	122	10	8.19
Iprobenfos	204	91.1	10	8.19
Iprodione	314	245	25	14.84
Iprodione	314	56	25	14.84
Iprodione	187	124	25	14.84
Isofenphos	213	185	15	10.72
Isofenphos	213	121	15	10.72
Isofenphos	185	121.1	15	10.72
Isofenphos-methyl	199	121	15	10.37
Isofenphos-methyl	199	65	15	10.37
Isoprothiolane	290	204	2	11.81
Isoprothiolane	290	118	10	11.81
Kresoxim-methyl	206	131	10	12.29
Kresoxim-methyl	206	116	5	12.29
Linuron	187	159	12	8.71
Linuron	187	124	31	8.71
Malathion	173.1	99	15	9.52
Malathion	173	127	4	9.52
Malathion	158	125	8	9.52
Mecarbam	329	160	3	10.74
Mecarbam	329	131	10	10.74

Mefenacet	193	137	12	16.27
Mefenacet	192	136	12	16.27
Mefenpyr-diethyl	252.8	190	20	14.69
Mefenpyr-diethyl	252.8	188.2	25	14.69
Mefenpyr-diethyl	252.8	163.2	25	14.69
Mepronil	119	91	15	13.28
Mepronil	119	65	15	13.28
Metalaxyl	206	162	20	8.98
Metalaxyl	206	132	5	8.98
Metazachlor	209	132.1	20	10.50
Metazachlor	133	117	25	10.50
Methacrifos	240	180	8	5.19
Methacrifos	208	180	4	5.19
Methidathion	145	85	5	11.15
Methidathion	145	58.1	15	11.15
Metribuzin	198.05	89	15	8.59
Metribuzin	198.05	82.1	20	8.59
Metribuzin	198	110	5	8.59
Mirex	271.9	237	15	16.22
Mirex	271.9	235	25	16.22
Mirex	271.9	116.9	40	16.22
Monolinuron	214	61	5	7.32
Monolinuron	126	98.9	15	7.32
Myclobutanil	179	152	6	12.15
Myclobutanil	179	125	14	12.15
Napropamide	271	128	5	11.68
Napropamide	128	72	5	11.68
Nuarimol	314	139	5	14.20
Nuarimol	235	139	15	14.20
Oxadiaxyl	163	132	5	13.08
Oxadiaxyl	163	117	15	13.08
Oxadiazon	301.9	175	13	12.03
Oxadiazon	174.9	112	15	12.03
Oxyfluorfen	361	300	15	12.16
Oxyfluorfen	252.05	196	20	12.16
Paraoxon-ethyl	149	102.1	20	9.01
Paraoxon-ethyl	108.9	81	10	9.01
Parathion-ethyl	291	109	10	9.81

Parathion-ethyl	291	81	10	9.81
Parathion-methyl	263	109.1	15	8.75
Parathion-methyl	263	79.1	30	8.75
PCB 028	258	186	15	8.60
PCB 028	258	150	27	8.60
PCB 052	292	255	12	9.31
PCB 052	292	220	27	9.31
PCB 052	289.9	220	23	9.31
PCB 101	325.9	291	12	11.29
PCB 101	325.9	256	34	11.29
PCB 101	325.9	254	34	11.29
PCB 118	327.9	255.9	25	12.77
PCB 118	325.9	256	27	12.77
PCB 118	325.9	254	27	12.77
PCB 138	361.8	289.9	25	13.96
PCB 138	359.8	325	15	13.96
PCB 138	359.8	290	27	13.96
PCB 153	361.8	289.9	25	13.29
PCB 153	359.8	325	15	13.29
PCB 153	359.8	290	27	13.29
PCB 180	395.8	323.9	22	15.56
PCB 180	393.7	359	12	15.56
PCB 180	393.7	324	27	15.56
Penconazole	248	192	15	10.57
Penconazole	161	125	25	10.57
Penconazole	159	123	25	10.57
Pendimethalin	252.1	162.1	10	10.52
Pendimethalin	252.1	161.2	20	10.52
Pentachloroanisole	264.8	235	25	7.12
Pentachloroanisole	264.8	143	15	7.12
Pentachlorobenzene	250	213	15	5.50
Pentachlorobenzene	249.9	214.9	25	5.50
Pentachlorobenzene	249.9	142	40	5.50
Permethrin	183.1	168.1	15	17.41
Permethrin	183	115.2	30	17.41
Phenthoate	274	125	20	10.80
Phenthoate	274	121	10	10.80
Phenylphenol ortho-(2-phenylphenol) (opp)	170	141.2	30	5.41

Phenylphenol ortho-(2-phenylphenol) (opp)	169	115	35	5.41
Phorate	260	75.2	5	6.75
Phorate	231	129	20	6.75
Phorate	75	47	5	6.75
Phosalone	182	138	5	15.98
Phosalone	182	111	15	15.98
Phosalone	182	75.1	30	15.98
Phosmet	160	133	20	15.05
Phosmet	160	105	15	15.05
Phosmet	160	77	30	15.05
Piperonyl-butoxide	175.9	145.2	15	14.39
Piperonyl-butoxide	175.9	117.1	20	14.39
Piperonyl-butoxide	175.9	103.1	25	14.39
Pirimicarb	238	166	10	8.25
Pirimicarb	166	96	15	8.25
Pirimiphos-ethyl	333.1	318.2	5	10.25
Pirimiphos-ethyl	318	109.1	35	10.25
Pirimiphos-methyl	305	290	10	9.32
Pirimiphos-methyl	305	180	5	9.32
Pirimiphos-methyl	290.1	125	25	9.32
Procymidone	283	255	10	10.95
Procymidone	283	96.1	10	10.95
Profenofos	337	267	10	11.88
Profenofos	337	188.1	30	11.88
Profenofos	208	63.1	35	11.88
Profluralin	318	199.2	20	7.49
Profluralin	318	54.9	20	7.49
Prometon	225	168	10	7.13
Prometon	210	168	5	7.13
Prometryn	241.2	183.9	5	8.96
Prometryn	241.2	111.2	5	8.96
Propanil	217	161	8	8.64
Propanil	161	99	25	8.64
Propazine	214	172	8	7.30
Propazine	214	94.4	25	7.30
Propiconazole	259	173	15	13.91
Propiconazole	259	69	12	13.91
Prosulfocarb	128	43.1	5	9.08

Prosulfocarb	128	41.1	5	9.08
Prothiofos	267	239	5	11.80
Prothiofos	162	63.1	30	11.80
prothiofos	309	239	20	11.80
Pyrazofos	232	204	10	16.79
Pyrazofos	221.1	149.1	15	16.79
Pyrazofos	221	193	10	16.79
Pyridaben	147	132	10	17.57
Pyridaben	147	117	20	17.57
Pyridaphenthion	340	199.2	5	14.95
Pyridaphenthion	340	108.2	15	14.95
Pyrifenox	262	227	10	10.62
Pyrifenox	171	100	25	10.62
Pyrimethanil	199	198	25	7.72
Pyrimethanil	198	156	25	7.72
Pyrimethanil	198	118	25	7.72
Pyriproxyfen	136	96	8	16.07
Pyriproxyfen	136	78	18	16.07
Quinalphos	157	129	15	10.87
Quinalphos	146.1	91.1	25	10.80
Quinalphos	146	118	15	10.80
Quintozene	295	237	20	7.60
Quintozene	237	119	30	7.60
Simazine	201.05	172.1	10	7.16
Simazine	186	91	5	7.16
Spiromesifen	370	272	15	14.79
Spiromesifen	370	254	15	14.79
Spiroxamine	100	72.2	10	8.68
Spiroxamine	100	43	10	8.68
Sulfotep	322.1	174	15	6.61
Sulfotep	322.1	146	25	6.61
Tebuconazole	252	127	25	14.19
Tebuconazole	250	125	25	14.19
Tebufenpyrad	333	276	5	15.37
Tebufenpyrad	333	171	20	15.37
Tecnazene	203	83.1	34	6.07
Tecnazene	202.9	143	20	6.07
Tecnazene	202.9	143	22	6.07

Tecnazene	202.9	83	25	6.07
Tefluthrin	177.1	137	20	7.87
Tefluthrin	177.1	127.1	20	7.87
Terbufos	231	174.9	10	7.52
Terbufos	231	128.9	25	7.52
Terbumeton	225	169	3	7.33
Terbumeton	169	154.2	5	7.33
Terbuthylazine	214.1	132	10	7.51
Terbuthylazine	214.1	104	20	7.51
Terbutryn	241.2	170.2	10	9.25
Terbutryn	185	170.1	5	9.25
Tetraconazole	336	218	15	9.97
Tetraconazole	336	204	30	9.97
Tetraconazole	336	164	25	9.97
Tetradifon	355.7	159	10	15.74
Tetradifon	353.7	159	10	15.74
Tetradifon	229	201	15	15.74
Tetramethrin I	164	106.9	10	15.13
Tetramethrin I	164	77.1	25	15.13
Thiobencarb	257	100	20	9.57
Thiobencarb	257	72	20	9.57
Thiometon	125	47	20	6.14
Thiometon	88	60	6	6.14
Tolclofos-methyl	265	250	15	8.84
Tolclofos-methyl	265	93	25	8.84
Tolylfluanid	238	137	10	10.66
Tolylfluanid	137	91	15	10.66
Triadimefon	208	181	5	9.86
Triadimefon	208	127	15	9.86
Triadimefon	181	111	10	9.86
Triadimenol	168	70	15	10.82
Triadimenol	128	65	20	10.82
Triazophos	257	162	5	13.40
Triazophos	161	134	5	13.40
Triazophos	161	106	10	13.40
Trifloxystrobin	222	130	8	13.85
Trifloxystrobin	131	116.1	15	13.85
Trifloxystrobin	116	89.1	20	13.85

Triflumizole	206	179.2	10	11.00
Triflumizole	206	143.9	30	11.00
Triflumizole	278	73	6	11.00
Trifluralin	306.1	160	25	6.47
**Trifluralin**	306	264	10	6.47
Triticonazole	235	217.2	5	15.89
Triticonazole	235	182.2	15	15.89
Vinclozolin	212	172	15	8.71
Vinclozolin	212	145	15	8.71

*Internal standard
